# Reimagining AHR in Cancer: From Environmental Sensor to Novel Immunomodulatory Therapeutic Target

**DOI:** 10.7150/ijbs.129455

**Published:** 2026-05-29

**Authors:** Yuanhao Peng, Yaxin Zhao, Yangying Zhou, Shuang Liu, Desheng Xiao, Yongguang Tao

**Affiliations:** 1 Hunan Key Laboratory of Cancer Metabolism, The Affiliated Cancer Hospital of Xiangya School of Medicine, Central South University/Hunan Cancer Hospital, Hunan Key Laboratory of Cancer Metabolism Changsha, 410013, Hunan, China.; 2 NHC Key Laboratory of Carcinogenesis, Cancer Research Institute, Xiangya School of Basic Medical Sciences, Central South University, Changsha, 410078, Hunan, China.; 3 Department of Critical Care Medicine, Xiangya Hospital, Central South University, Changsha, 410078, Hunan, China.; 4 Department of Oncology, Xiangya Hospital, Central South University, Changsha, 410078, Hunan, China.; 5 Department of Oncology, Institute of Medical Sciences, National Clinical Research Center for Geriatric Disorders, Xiangya Hospital, Central South University, Changsha, 410078, Hunan, China.; 6 Department of Pathology, Xiangya Hospital, Central South University, Changsha, 410078, Hunan, China.

**Keywords:** AHR, cancer, mitochondrial metabolism, epigenetics, cell fate, tumor immunity

## Abstract

The aryl hydrocarbon receptor (AHR) is an environmental sensor in mammals and a ligand-dependent, highly conserved transcription factor. It belongs to the basic helix-loop-helix family of transcription factors and is the only known ligand-activated member within this family. Previous studies have revealed its significant roles in physiological regulation, metabolic homeostasis, and tumorigenesis. *AHR* governs transcriptional regulation and epigenetic modifications through diverse mechanisms and plays an important role in various types of cancer. In this review, we introduce the history and structure of *AHR* and summarize its modes of action via canonical and non-canonical pathways. We elaborate on the distinct impact of *AHR* on mitochondrial metabolism, epigenetics, as well as cell death and fate. Furthermore, we systematically discuss the relationship between *AHR* and tumor immunity. Finally, we explore the prospects of *AHR* in the tumor microenvironment, cancer immunity and therapy, and its potential as an immunotherapeutic target, along with current achievements in drug development targeting AHR. These research findings may provide insights into the relationship between AHR and its regulated molecules and pathways in cancer, as well as mechanisms for cancer treatment and intervention.

## Introduction

The aryl hydrocarbon receptor (AHR), an evolutionarily conserved ligand-activated transcription factor, maintains a predominantly cytoplasmic localization in its unbound state 1. As a distinctive member of the Basic Helix-Loop-Helix/Per-Arnt-Sim (BHLH-PAS) superfamily, AHR exhibits unique ligand-dependent activation properties not observed in other family members. While initial investigations primarily characterized its role in the detoxification of environmental pollutants, contemporary research has revealed its capacity to integrate diverse signaling inputs from environmental toxicants, dietary constituents, microbiome-derived metabolites, and endogenous ligands 2-4. This multimodal signaling capability establishes AHR as a critical regulator of fundamental biological processes, including cellular differentiation, immunomodulation, and metabolic homeostasis maintenance. This expanded functional paradigm has revitalized interest in targeting *AHR* for therapeutic intervention in malignancies, inflammatory disorders, and autoimmune conditions. Notably, increasing evidence has revealed *AHR* activation by various diet-derived phytochemicals present in common food sources, suggesting potential mechanistic links between nutritional components and AHR-mediated physiological regulation 5-7. These findings underscore the need for systematic investigations of the pleiotropic functions of *AHR*, particularly its context-dependent roles in human pathophysiology.

AHR has dual functions in tumor biology, exhibiting context-dependent oncogenic and tumor-suppressive roles. Pathologically elevated *AHR* expression has been documented across multiple malignancies, including glioma, gastrointestinal carcinomas, breast cancer, and non-small cell lung cancer (NSCLC). Mechanistically, *AHR* participates in tumor evolution by regulating the characteristics of cancer stem cells, epithelial‒mesenchymal transition (EMT), and remodeling of the immune microenvironment 8, 9. Given its multifaceted role in tumor biology, *AHR* has emerged as a key driver in the development of combinatorial therapeutic strategies., with small molecule inhibitors (e.g., IK-175 and BAY 2416964) currently undergoing clinical evaluation in solid tumors 10, 11. Notably, the dual IDO1/TDO2 inhibitor epacadostat combined with anti-PD-1 immunotherapy has completed phase III assessment in advanced melanoma, highlighting the translational relevance of AHR 12. Critically, AHR-mediated biological responses exhibit strict ligand specificity and tissue-selective activity, demonstrating context-dependent bidirectional regulation. This functional plasticity is governed by two principal determinants: the pharmacological properties of the ligand (agonist versus antagonist) and spatiotemporal exposure parameters. In tumors, metabolic reprogramming within the tumor microenvironment (TME), such as activation of the tryptophan (Trp)-kynurenine (Kyn) axis, dynamically alters AHR activation status 13, 14. Furthermore, diet-derived AHR ligands from cruciferous vegetables (e.g., indole-3-carbinol (I3C)) demonstrate epigenome-modifying potential, suggesting that nutritional modulation may influence tumor susceptibility landscapes, a finding with profound implications for precision dietary interventions in oncology 15, 16.

This article presents a comprehensive review of the major roles of AHR and its signaling pathways in cancer stem cells and the tumor immune microenvironment. We highlight the importance of AHR in cancer biology and therapy and explore its potential as a key area for further research and clinical development. By integrating existing research data, this study aims to provide novel perspectives and a theoretical foundation for the application of AHR in cancer treatment.

### The origin and structure of AHR

The AHR represents an evolutionarily ancient protein conserved across animal phylogenetics, with ancestral forms dating back more than 550 million years. AHR and its homologous proteins exhibit broad phylogenetic distributions and are preserved in nematodes, mollusks, Drosophila, and all major chordate lineages, including vertebrates 8. Through evolutionary processes, this receptor has acquired pleiotropic characteristics, exhibiting species specific, developmental stage dependent, and cell type-selective functionalities. The discovery of 2,3,7,8-tetrachlorodibenzo-p-dioxin (TCDD) as a potent AHR ligand originated from observations of its ability to induce aryl hydrocarbon hydroxylase activity and upregulate the cytochrome P450 family 1 subfamily A member 1 (CYP1A1) expression in mammalian systems, which subsequently stimulated substantial research interest in toxicology and pharmacology 9. Subsequent investigations in the life sciences have significantly expanded our understanding of the pathophysiological roles of AHR, revealing its functional dichotomy, a characteristic that manifests dual regulatory roles in vital biological processes. Current evidence highlights its context-dependent effects across multiple physiological systems, necessitating continued mechanistic exploration of this multifunctional receptor.

In the unactivated state, cytoplasmic AHR forms a stable macromolecular complex consisting of two heat shock proteins90 (HSP90) that interact with AHR, an X-associated protein 2 (XAP2) and a p23 cochaperone 17. Cryoelectron microscopy studies of the human cytoplasmic complex revealed that AHR and HSP90 adopt a closed conformation, forming a stable binary structure. In this configuration, the PAS-A-PAS-B junction traverses the HSP90 lumen, with the PAS domains positioned on opposing sides of the HSP90 molecule. This spatial arrangement prevents AHR degradation while maintaining high ligand-binding affinity 18. Mechanistically, HSP90 maintains AHR in an inactive state by masking its nuclear localization signal. Within this complex, XAP2 functions as a scaffolding protein that stabilizes the AHR structure, particularly its Transactivation domain region 19, 20. Concurrently, p23 interacts with HSP90 via multiple hydrogen bonds and salt bridges, effectively inhibiting its nuclear translocation and enhancing its complex stability. The PAS-B domain serves as the primary ligand-binding domain, functioning as a sensor for environmental and physiological signals. Structural analyses revealed that the PAS-A domain lacks internal cavities suitable for ligand binding and is occluded by bulky aromatic residues. This observation suggests that the PAS-A domain primarily mediates protein‒protein interactions, including AHR‒ARNT heterodimerization and increased DNA binding 21. Following ligand-induced AHR activation, the HSP90 molecular chaperone undergoes a conformational transition to an open state, thereby unmasking the NLS and initiating nuclear translocation. This activation cascade involves sequential molecular events: dissociation of HSP90, followed by replacement of XAP2 and p23 with the aryl hydrocarbon receptor nuclear translocator (ARNT), culminating in the formation of a transcriptionally active AHR‒ARNT heterodimer that exhibits increased DNA-binding affinity 22. ARNT, a nuclear dimerization partner for multiple transcription factors (also known as HIF-β.), including hypoxia-inducible factor (HIF) and estrogen receptor (ER) 23, enables the heterodimer to recognize xenobiotic response elements (XREs) in target gene promoters for transcriptional regulation. Notably, emerging evidence challenges the classical activation model. Human cellular studies have demonstrated that AHR nuclear translocation can occur without complete HSP90 dissociation 24. Furthermore, nuclear accumulation does not necessarily correlate with transcriptional activation, as AHR exhibits constitutive nucleocytoplasmic shuttling independent of exogenous ligands—a process devoid of transcriptional activation capacity. Intriguingly, the nuclear AHR population maintains stability through mechanisms independent of DNA binding or ARNT interactions, suggesting that ligand-induced conformational changes may promote nuclear retention while preventing nuclear export through autonomous regulatory mechanisms 25.

### Mechanisms of action of AHR

**Figure 1:**
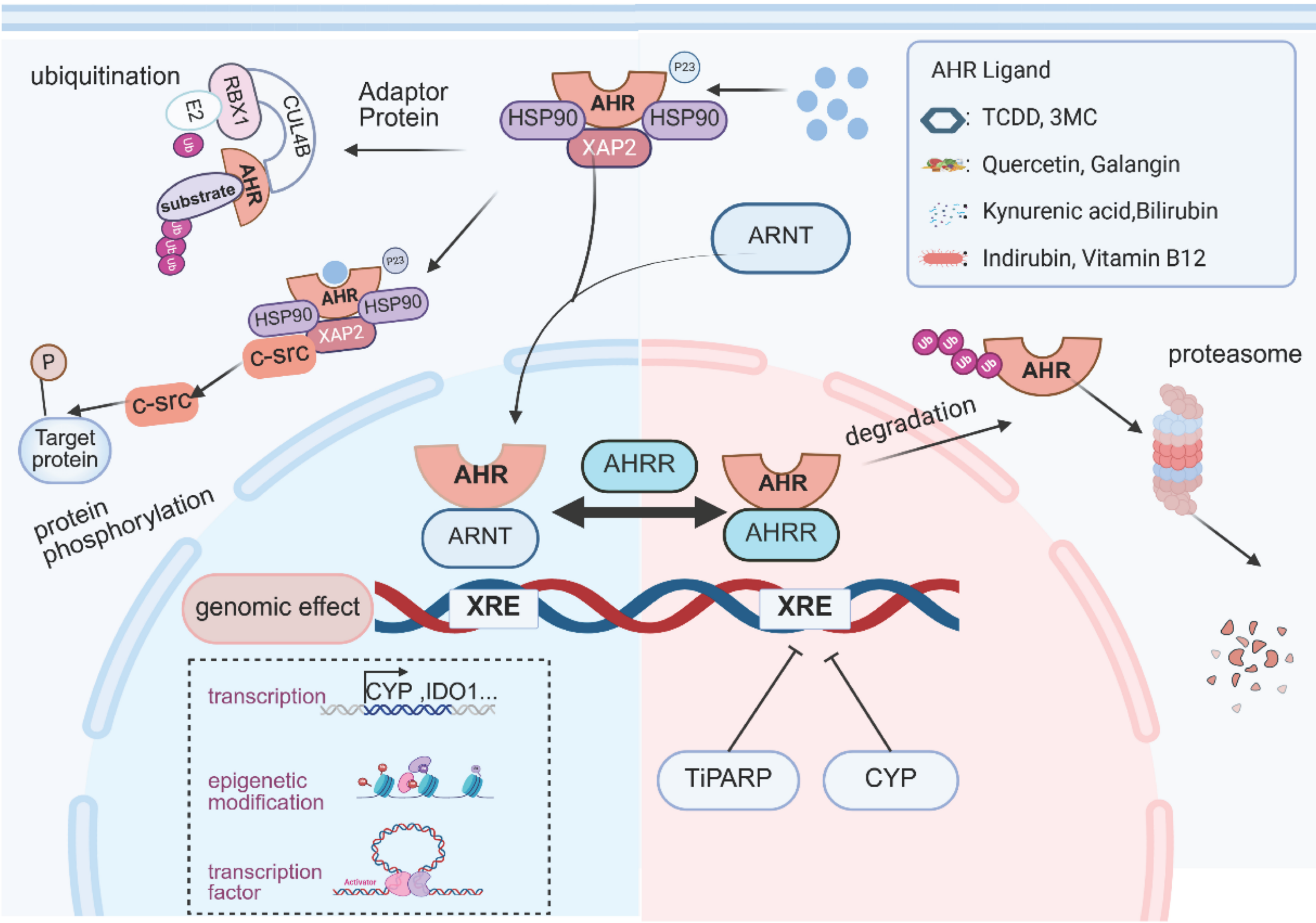
** Mechanism of AHR Signaling.** The inactive form of AHR resides in the cytosol as a complex with chaperone proteins (HSP90, XAP2, and p23). Upon binding to ligands derived from the gut microbiota, host metabolism, dietary components, or environmental sources, AHR undergoes conformational changes and initiates nuclear translocation. Within the nucleus, the AHR complex recruits ARNT, replacing HSP90 to form an AHR-ARNT heterodimer. This heterodimer binds to XRE sequences, directly activating downstream gene transcription. In addition to canonical transcriptional regulation, AHR exerts indirect genomic control through epigenetic modifications and interactions with other transcription factors. Nongenomic regulatory roles include the assembly of E3 ubiquitin ligase complexes and the activation of protein kinases. To ensure precise modulation of AHR signaling, a multilayered negative feedback system tightly regulates activation duration and intensity. The key mechanisms include the following: CYP family enzymes metabolize AHR ligands, competitive inhibition of ARNT by AHRR, nuclear export mediated by TiPARP, and degradation of AHR via the ubiquitin‒proteasome pathway. This dynamic regulation maintains the homeostasis of AHR-mediated biological responses. This image was created using BioRender.com.

Upon ligand binding, AHR functions as a transcriptional regulator that modulates gene expression through three coordinated mechanisms: direct promoter engagement at XREs, selective recruitment of transcriptional coactivators and corepressors, and dynamic crosstalk with intracellular signaling pathways (Figure 1) 3, 4, 26.

#### Canonical (XRE-dependent) Genomic Signaling

AHR functions as a ligand-activated transcription factor that dimerizes with ARNT to recognize and bind specific dioxin response elements (DREs) or XREs within target gene promoters. These regulatory elements characteristically contain a conserved 5′-GCGTG-3′ core motif, typically positioned within 300 bp upstream of transcription start sites in promoter regions 27, 28. The AHR/ARNT heterodimer binding to XRE induces chromatin remodeling in promoter regions, facilitating the recruitment of RNA polymerase II preinitiation complexes to initiate transcription. This molecular cascade activates key xenobiotic-metabolizing enzymes, including CYP1A1, CYP1A2 and CYP1B1 29, 30. In addition to this canonical transcriptional activation, AHR orchestrates epigenetic regulation through three distinct mechanisms: chromatin architectural control via brahma/SWI2-related gene 1 (Brg1)-mediated remodeling and steroid receptor coactivator-1 complex recruitment 31; transcriptional derepression through competitive displacement of histone deacetylase (HDAC) complexes; and noncoding genome regulation involving retrotransposon silencing, microRNA modulation, and long noncoding RNA (lncRNA) interactions 32.

#### Noncanonical (XRE-independent) Genomic Signaling

In addition to its canonical transcriptional regulation, AHR exhibits cross-regulatory capacity through the modulation of other transcription factors. Studies have shown that AHR antagonizes estrogen signaling by modulating ER activity. In rats chronically exposed to AHR ligands, the incidence of uterine and mammary tumors was lower than that in control animals 33. Mechanistically, AHR directly regulates ER through an interaction site located within the P/S/T region of its transactivation domain 34. Furthermore, the AHR/ARNT complex competes with ER for nuclear receptor co-regulators such as ERAP140 and SMRT, thereby further antagonizing estrogen signaling 35. And AHR also interacts with the core clock gene Basic helix-loop-helix ARNT like 1 (BMAL1); the structural homology between these two transcription factors enables them to heterodimerize through their PAS domains 36. Activation of AHR has been shown to disrupt CLOCK-BMAL1 complex activity, leading to suppressed Per1 expression and subsequent circadian disruption 37.

#### Nongenomic AHR Signaling

Apart from genomic regulation, AHR exerts noncanonical control over cellular processes through nongenomic signaling mechanisms. AHR has been reported to be involved in scaffolding Cullin 4B (CUL4B)-based E3 ubiquitin ligase complexes, facilitating the proteasomal degradation of nuclear receptors, including estrogen receptor α, androgen receptor, and peroxisome proliferator-activated receptor γ 38. For instance, a study on bladder cancer revealed that AHR suppresses STING signaling through a negative feedback mechanism. Interestingly, activation of AHR did not alter STING mRNA levels. Further investigation revealed that AHR functions as an adaptor protein, recruiting the CUL4B-RBX1 E3 ligase complex to mediate K48-linked ubiquitination of STING at lysine 236, thereby promoting its proteasomal degradation. This results in reduced production of type I interferons and impaired T cell-dependent antitumor immunity 39. The nongenomic effects of AHR involve protein kinase activation. In the cytoplasm, AHR interacts with non-receptor tyrosine kinases such as steroid receptor coactivator (SRC). Notably, c-SRC has been reported to serve dual functions as both an AHR chaperone and a signaling partner 40. On ligand-induced AHR activation, c-SRC dissociates from the cytoplasmic AHR/HSP90/c-SRC complex and subsequently phosphorylates multiple cellular targets 41, 42.

#### Negative feedback regulation of AHR activation

The regulation of activated AHR is a dynamic process: Upon initiating the transcriptional activation of target genes, AHR levels progressively decrease. The receptor is rapidly exported from the nucleus to the cytoplasm for proteasomal degradation, although the precise degradation mechanisms remain incompletely understood. Current evidence suggests that nuclear-to-cytoplasmic export is a prerequisite for the ubiquitination-mediated 26S proteasomal degradation of AHR 27,28. HSP90 dissociation appears to be critical for proteasome recognition, but emerging data implicate E3 ubiquitin ligases in activated AHR degradation 29. To constrain AHR-mediated transcriptional programs, organisms employ sophisticated negative feedback mechanisms that regulate both the duration and intensity of AHR activation. Ligand-activated AHR induces the transcription of cytochrome *CYP1A1*, *CYP1B1*, *aryl hydrocarbon receptor repressor (AHRR)*, and TCDD-inducible poly-ADP-ribose polymerase (TiPARP), which collectively suppress AHR signaling through multiple mechanisms.

CYP1A1 catalyzes the oxidative metabolism of AHR ligands, facilitating their clearance and effectively terminating AHR signaling 43, 44. Murine studies revealed that constitutive *CYP1A1* expression depletes endogenous AHR ligand pools, inducing quasi-AHR-deficient states. Intestinal epithelium-specific CYP1A1 overexpression ablates AHR-dependent group 3 innate lymphoid cells and T helper 17 (Th17) populations, increasing susceptibility to enteric pathogens—a phenotype rescued by exogenous ligand supplementation 45. Importantly, CYP activity modulation (via UVB exposure, hydrogen peroxide, dietary substrates, or epigenetic regulation) may induce ligand-specific degradation, potentially confounding the interpretation of CYP inhibitors as false AHR agonists 46.

AHRR, a BHLH/PAS family protein, exhibits substantial N-terminal homology with AHR, which contains conserved DNA-binding BHLH and PAS-A domains but lacks the C-terminal ligand-binding PAS-B domain and glutamine-rich transactivation domain characteristic of AHR 47. First, AHRR competes with AHR for ARNT heterodimerization, thereby preventing XRE binding and subsequent target gene activation 48. Second, AHRR recruits corepressor complexes (including histone deacetylases) to XRE-containing promoters, inducing chromatin condensation that impedes transcription factor accessibility 49. Third, structural dissection studies have revealed an ARNT-independent repression mechanism mediated through the AHRR N-terminal region. This "transrepression" mode operates via protein‒protein interactions rather than disrupting AHR‒ARNT complex formation or DNA binding. Supporting evidence includes the following: ARNT overexpression fails to rescue AHRR-mediated transcriptional suppression; C-terminal truncation mutants retain full inhibitory capacity 50.

TiPARP suppresses AHR target gene transcription through two distinct mechanisms: direct DNA binding or anchoring interaction with AHR, both of which require intact zinc finger and catalytic domains. Additionally, TiPARP overexpression enhances proteolytic degradation of AHR via either direct ADP-ribosylation or indirect modulation through unidentified intermediaries, thereby inhibiting AHR-mediated transactivation 51. Emerging evidence indicates that TiPARP regulates ligand-induced AHR nuclear export. In TiPARP knockout models, TCDD-activated AHR accumulates in the nucleus 52. Further mechanistic studies reveal that TiPARP mediates mono-ADP-ribosylation of the AHR nuclear export signal motif, facilitating AHR export from the nucleus to the cytoplasm for subsequent degradation 53.

#### Ligands of AHR

As the understanding of AHR has increased, AHR research has gradually expanded from toxicology to all aspects of physiology, and the sources of AHR ligands have increased, ranging from strong-affinity exogenous agonists such as planar halogenated polycyclic hydrocarbons and polycyclic aromatic hydrocarbons (PAHs), as represented by TCDD, to dietary sources, the formation of free radicals, and enzyme activities in the host and commensal flora, among others (Table 1).

Environmental pollutants, exemplified by TCDD, exhibit high affinity for AHR and possess potent toxicity. TCDD can enter the human body through direct exposure or bioaccumulation. TCDD induces and binds to CYP1A2 in the liver, a process that restricts cytochrome P450-mediated metabolism, leading to hepatic sequestration and prolonged retention, thereby making it difficult to eliminate from the body. Consequently, TCDD has a half-life of several years in humans 81, 82. This bioaccumulation results in sustained AHR activation, which in turn triggers a range of toxicological effects, including cancer, reproductive disorders, hepatobiliary injury, and neurodegenerative diseases, necessitating stringent regulatory control 83-85. In contrast, dietary AHR ligands are more commonly encountered in daily life and include cruciferous vegetables (such as Brussels sprouts, cabbage, and cauliflower), coffee, and various spices 86-90. These compounds generally exhibit lower affinity for AHR. Taking cruciferous vegetables as an example, they are rich in indole glucosinolates. When the plant tissue is chewed or cut, these glucosinolates are converted by plant myrosinase into I3C. This compound is considered a pro-ligand that undergoes further chemical transformation under the acidic conditions of the stomach to generate a variety of functional AHR ligands, including DIM, alkyl- and chlorine-substituted 3,3′-diindolylmethanes, linear and cyclic di-, tri-, and tetraindoles, and condensed derivatives such as indolo[3,2-b] carbazole. Among these, ICZ exhibits particularly high affinity for AHR 88, 91. Additionally, various structurally diverse and relatively safe AHR ligands are present in the diet, such as quercetin, curcumin, and resveratrol 90. Despite the AHR-activating potential of various foods, their complex composition and the lack of knowledge regarding their efficacy at doses relevant to human intake remain major limitations. Therefore, while dietary AHR ligands warrant systematic evaluation as potential intervention targets, they nevertheless represent a promising direction for future research.

Most endogenous physiological activation of AHR originates from host- and microbiota-mediated Trp metabolism. As an essential amino acid that cannot be synthesized by the human body, Trp must be obtained through dietary sources such as meat, eggs, fish, and dairy products 92. Approximately 95% of Trp is catabolized via the Kyn pathway, primarily through two rate-limiting enzymes: tryptophan 2,3-dioxygenase (TDO) and indoleamine 2,3-dioxygenase (IDO), which convert Trp into Kyn. Subsequently, kynurenine aminotransferase (KAT) further converts Kyn into kynurenic acid (KynA). Both Kyn and KynA are key effector molecules in activating AHR 92, 93. Through AHR activation, these metabolites influence a broad range of physiological and pathological processes, including cancer, aging, and immunity 94-97. For example, IDO1 and TDO expression levels correlate with glioma grade, and the IDO1/TDO-Kyn-AHR-AQP4 axis has been shown to promote glioblastoma progression 98. In tuberculosis, IDO1 is upregulated in inflammatory macrophages, leading to increased Kyn production. Kyn-mediated AHR activation suppresses JAK-STAT1 signaling, reduces the secretion of the chemokines CXCL9 and CXCL10, impairs T-cell infiltration, and thereby delays T-cell immune responses 99. In addition to host metabolism, the gut microbiota also contributes significantly to the pool of endogenous AHR ligands by directly converting Trp into a wide array of metabolites, including indole and its derivatives such as indole-3-aldehyde, indole-3-acetic acid, and indole-3-propionic acid. These microbial metabolites play critical roles in maintaining intestinal epithelial renewal, barrier integrity, and gut immune homeostasis 100-102.

Despite significant advancements in understanding AHR signaling, the molecular mechanisms underlying ligand binding specificity and subsequent activity regulation remain elusive, primarily due to incomplete structural characterization of AHR domains. Recent progress combining homology modeling of BHLH-PAS proteins with cryo-EM technological breakthroughs has enabled structural resolution of both the cytoplasmic AHR complex and its PAS-B domain. In contrast to conventional models, Dai *et al*. (2022) demonstrated through Drosophila AHR PAS-B domain analysis that ligand binding (e.g., α-naphthoflavone [αNF]) induces conformational changes distal to the AHR/ARNT dimerization interface. Subsequent luciferase reporter assays revealed that these structural rearrangements do not influence murine AHR transcriptional activity, suggesting that ligands function primarily as nuclear translocation switches rather than as transcriptional modulators posttranslocation 17. Furthermore, Tagliabue *et al*. (2019) revealed distinct interaction patterns between various ligands and specific residues within the AHR ligand-binding pocket, which may lead to differential effects on AHR conformational changes as well as interactions with protein chaperones that may propagate downstream of the AHR signaling pathway 103. Further efforts are still needed in the future to elucidate the structure of AHR to identify new AHR ligands and develop efficient anticancer AHR drugs, as well as to further elucidate the proposed mechanism of ligand-dependent AHR transformation, such as the process of HSP90 transition to the open conformation, and how the different binding modes of ligands can affect the transcription of downstream genes.

### AHR and cancer

**Figure 2:**
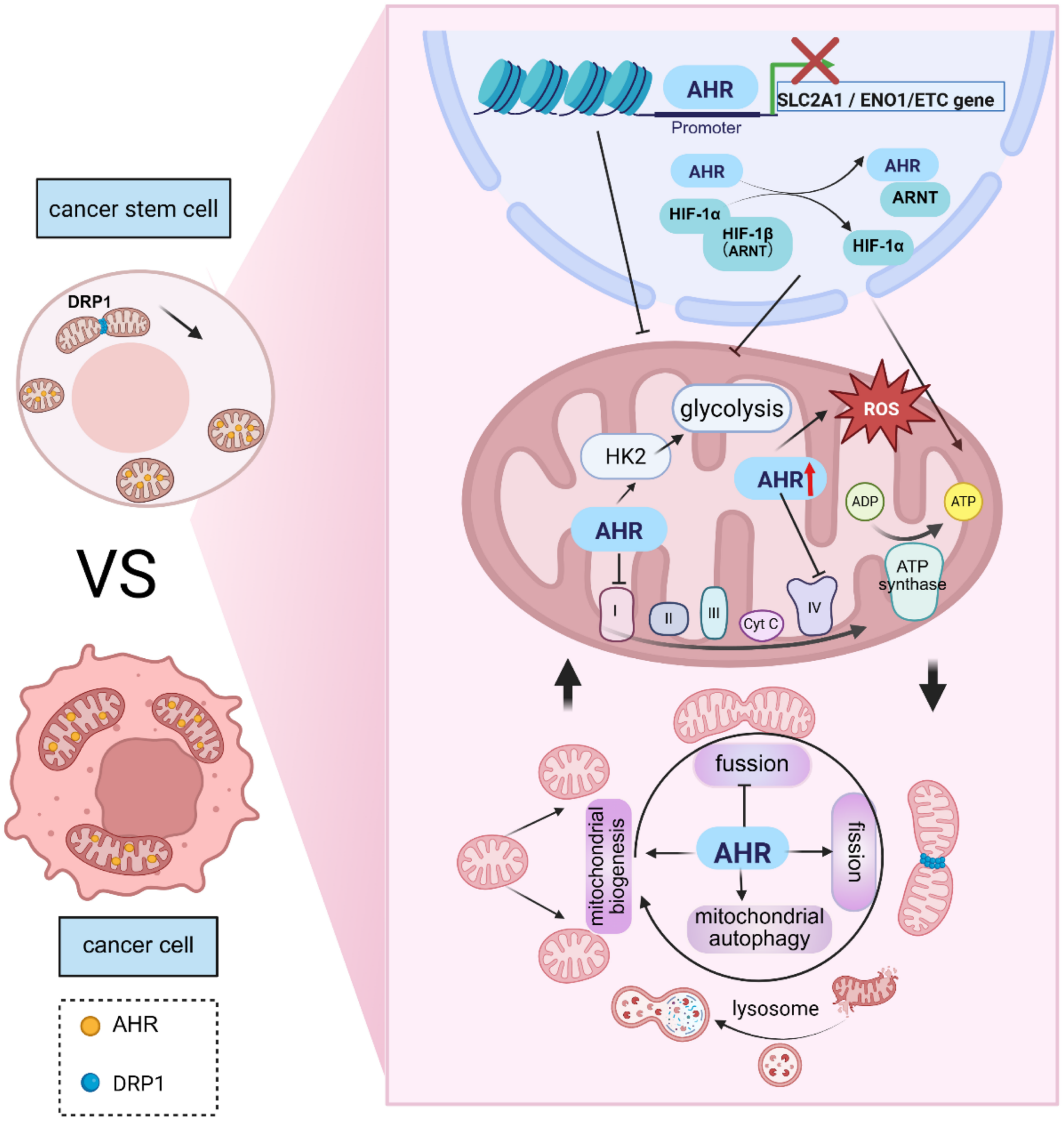
**AHR as a Key Regulator of Mitochondrial Function in Cancer Stem Cells.** AHR not only localizes to the nucleus to exert canonical transcriptional regulation but also prominently colocalizes with the mitochondrial matrix and outer membrane, suggesting its direct involvement in mitochondrial quality control systems. Compared with differentiated cancer cells, CSCs exhibit unique mitochondrial morphology characterized by a smaller size, rounded shape, and interconnected network structures—features critical for maintaining their stemness. AHR has dual regulatory effects on glycolysis and OXPHOS. Mechanistically, AHR suppresses glycolysis by inhibiting the transcription of SLC2A1 and ENO1 while competing with HIF1A for binding to HIF-1β, thereby destabilizing the HIF1 complex. Conversely, AHR transcriptionally activates HK2, a key glycolytic enzyme, significantly increasing glycolytic flux. Notably, AHR activation inhibits mitochondrial respiratory chain activity, represses 20 ETC-related genes and directly interacts with mitochondrial proteins to suppress complex I and complex V activity. Furthermore, AHR dynamically regulates mitochondrial dynamics in CSCs—promoting fission and mitophagy while inhibiting fusion—to fine-tune metabolic adaptation and maintain stemness. This image was created using BioRender.com.

Cancer stem cells (CSCs) are a subpopulation of undifferentiated cells with high self-renewal ability in tumor tissues. The unique biological properties of CSCs establish them as central drivers of tumor malignancy 104, 105. Through their robust self-renewal and tumorigenic potential, CSCs not only promote tumor invasion and metastasis but also orchestrate key pathological processes, including chemoresistance and disease recurrence, via dynamic modulation of the tumor microenvironment 106-109. The AHR has recently emerged as a critical molecular interface connecting environmental signals with CSC maintenance 110, 111. By binding diverse endogenous and exogenous ligands, AHR modulates downstream signaling pathways to regulate CSC biology through stemness-associated gene expression, epigenetic reprogramming, cell death mechanisms, and metabolic plasticity. This section systematically examines the mechanistic role of AHR in CSC pathophysiology and evaluates its potential as a therapeutic target through four integrated dimensions: stemness regulation and mitochondrial function, epigenetic remodeling, and cell death pathways (Figure 2).

#### AHR and stemness regulation

Octamer-binding transcription factor 4 (OCT4), SRY-box transcription factor 2 (SOX2), Nanog, and Kruppel-like factor 4 constitute core pluripotency factors essential for maintaining the self-renewal, proliferation, and differentiation capacities of CSCs 112, 113. These transcriptional regulators are significantly upregulated in CSCs across diverse malignancies, including breast 114, 115, pancreatic 116, and lung 117 cancers. Elevated expression of these factors not only enhances tumor-initiating potential, chemoresistance, and metastatic competence 118-120 but also correlates negatively with clinical outcomes, which are associated with poor prognosis and reduced patient survival 121-123. Nacarino-Palma *et al*. (2021) demonstrated that constitutive AHR deficiency increases the expression of stem cell regulators (e.g., OCT4 and SOX2), promoting stem cell expansion and driving non-small cell lung cancer pathogenesis 124. Cheng *et al*. (2015) established an inverse correlation between AHR and OCT4 expression across human embryonic stem cells, embryonic carcinoma cells, tumor cell lines (HeLa, HepG2, U87, HT-29, and MCF-7), and nontumor lines (HUVEC, LO2, and 293T). Mechanistically, AHR binds the OCT4 promoter to repress transcriptional activity 125.

In contrast, evidence indicates that AHR activation may potentiate cancer stemness. Exogenous administration of Kyn can upregulates OCT4 and SOX2 expression, increasing the tumor sphere-forming capacity of breast cancer cells 126. Furthermore, AHR stabilizes the SOX2 protein by suppressing its ubiquitination via protein kinase A pathway activation, consequently augmenting cancer stemness in small cell lung cancer (SCLC) and correlating with poor clinical outcomes in SCLC patients^127^. AHR plays a dual role in regulating stemness gene expression in CSCs. It can suppress the self-renewal and tumorigenic potential of CSCs by downregulating the expression of stemness genes such as OCT4 and SOX2. Conversely, AHR activation may enhance CSC stemness by upregulating these genes or inhibiting their ubiquitination. This dual nature likely depends on the AHR ligand type, heterogeneity of the tumor microenvironment, and cross-regulation of signaling pathways. Future research should investigate the role of AHR in diverse tumor microenvironments and its regulatory mechanisms in maintaining CSC stemness.

#### AHR and EMT

EMT represents a critical biological process wherein polarized epithelial cells lose intercellular adhesion properties and acquire mesenchymal phenotypes 128. This dynamic plasticity significantly impacts the clinical outcomes of malignant tumors by increasing tumor cell invasiveness, inducing apoptosis resistance, and conferring therapeutic tolerance 129-131. Notably, EMT not only constitutes a core regulatory mechanism for CSCs but also endows disseminated tumor cells with hybrid epithelial‒mesenchymal stemness properties, rendering them the most aggressive and chemotherapy-resistant metastatic precursors 132-134. Recent studies have demonstrated that AHR plays pivotal roles in EMT regulatory networks. Li *et al*. (2017) 135 revealed that cytoplasmic AHR exerts EMT-suppressive effects through accelerated vimentin degradation, whereas ligand-mediated AHR nuclear translocation may constitute a critical molecular switch for EMT activation. In lung cancer models, sustained benzopyrene (BaP)-induced AHR activation induces canonical EMT phenotypic conversion, characterized by E-cadherin suppression and significant N-cadherin upregulation 136. EMT initiation depends on the integration of microenvironmental signals (TGF-β, Wnt, and Notch), with the TGF-β axis occupying central regulatory dominance: the canonical Smad pathway regulates mesenchymal phenotype-associated gene expression via SMAD complex nuclear translocation, whereas noncanonical pathways cooperatively promote EMT through posttranslational modifications, including phosphorylation/ubiquitination 137-141. In SHH medulloblastoma, AHR maintains tumor cell differentiation by inhibiting SMAD3 phosphorylation; its deficiency causes aberrant TGF-β/SMAD3 pathway activation and undifferentiated phenotypes 142. Conversely, during colitis-associated carcinogenesis, TGF-β synergizes with IL-6 to induce AHR expression and promote IL-22 secretion by Th17 cells through ligand-dependent activation, thereby remodeling the tumor immune microenvironment 143, 144. In glioma research, Gramatzki *et al*. (2009) discovered tissue-specific dual regulatory characteristics in AHR-TGF-β signaling crosstalk: AHR inhibition downregulates the TGF-β/Smad pathway in human glioblastoma cells, reducing proliferation and invasiveness; in contrast, AHR negatively regulates TGF-β signaling in nonneoplastic astrocytes 145. However, the AHR-TGF-β relationship transcends unidirectional regulation, with accumulating evidence supporting dynamic bidirectional crosstalk. Miret *et al*. (2016) 146 demonstrated that the AHR activator hexachlorobenzene rapidly triggers c-Src activation through AHR, increasing the phosphorylation of TGF-β1 downstream effectors (Smad3, JNK, and ERK1/2). Conversely, when TGF-β1 concentrations reach threshold levels in the TME, an inhibitory feedback loop suppresses AHR expression. This homeostatic imbalance ultimately promotes breast cancer invasion and progression. Current evidence reveals that AHR and the TGF-β/EMT axis establish intricate regulatory networks through positive/negative feedback loops. AHR-TGF-β interaction nodes may represent novel therapeutic targets to overcome EMT-associated treatment resistance. However, selection between AHR agonists/antagonists requires the consideration of specific TME characteristics for precise modulation of the context-dependent tumor-suppressive or oncogenic functions of AHR.

### AHR and mitochondrial metabolism

Emerging research reveals that mitochondria in CSCs function beyond classical "powerhouses," instead of operating as regulatory hubs that govern CSC fate through multiple mechanisms 147, 148. Compared with differentiated cancer cells, CSCs exhibit distinct mitochondrial morphology characterized by small, globular structures with a networked organization. This architectural specialization confers metabolic particularities such as succinate and L-2-hydroxyglutarate accumulation—metabolites that sustain CSC self-renewal by inhibiting histone lysine demethylase activity 149. DRP1 knockdown transforms mitochondrial morphology from fragmented spheres to elongated forms in oral squamous cell carcinoma stem cells, concomitant with increased α-ketoglutarate (α-KG) levels that drive TCA cycle flux and histone modification, consequently suppressing stemness while increasing ferroptosis susceptibility 150. The metabolic phenotypes of CSCs vary among malignancies, with subsets exhibiting either glycolytic dominance or oxidative phosphorylation (OXPHOS) dependence 151. In prostate cancer, hepatocellular carcinoma, osteosarcoma, lung cancer, nasopharyngeal carcinoma, and other solid tumors, CSC subpopulations primarily utilize aerobic glycolysis to generate ATP and metabolic intermediates that support stemness maintenance. Glycolysis inhibitors, which represent promising therapeutic targets, have demonstrated significant anti-CSC efficacy 152. Conversely, glioblastoma, pancreatic ductal adenocarcinoma, and ROS-low quiescent leukemia stem cells display profound OXPHOS dependence. Notably, forced OXPHOS utilization in PDAC cells enriches CSCs, as evidenced by elevated CSC biomarker expression, increased tumorigenic potential, and increased immune evasion capacity 153. Regardless of their metabolic configuration, mitochondria remain pivotal regulators of CSC functionality.

CSC**s** typically reside within hypoxic tumor niches and exhibit elevated OXPHOS capacity with low reactive oxygen species (ROS) levels and robust antioxidant defense systems that collectively sustain self-renewal and stemness 154, 155. Mitochondrial dysfunction increases ROS generation, effectively suppressing self-renewal while triggering CSC differentiation 156. Furthermore, mitochondrial subcellular localization and dynamics—including fission/fusion processes—are essential for maintaining CSC stemness. Asymmetric mitochondrial distribution influences daughter cell fate: Progeny receiving fewer perinuclearly localized aged mitochondria retain stem-like properties. Inhibiting mitochondrial fragmentation disperses senescent mitochondria throughout the network, causing daughter cells to lose their stem cell characteristics 157. Through their unique morphology, metabolic phenotypes, and subcellular positioning, mitochondria serve as key regulators of CSC fate. Their dynamic modulation not only impacts CSC self-renewal and therapy resistance but also affects tumor aggressiveness and treatment response. Elucidating mitochondrial regulatory mechanisms in CSCs will provide a theoretical foundation for developing metabolism-targeted interventions against CSC populations.

Multiple studies have identified AHR as a key molecule linking CSC metabolic phenotypes to mitochondrial function regulation. Previous research has demonstrated that AHR colocalizes with mitochondria in specific cell types 158, where its cytoplasmic binding partners AIP and HSP90 interact with the mitochondrial outer membrane translocase complex to facilitate the mitochondrial import of proteins lacking classical mitochondrial targeting sequences (MTSs), including AHR. Notably, TCDD-induced mitochondrial dysfunction may involve aberrant degradation of mitoAHR 159. This subcellular colocalization suggests direct AHR involvement in the regulation of mitochondrial function. The following sections systematically delineate AHR regulatory mechanisms across three dimensions: mitochondrial metabolic control, quality surveillance systems, and stress responses.

#### Mitochondrial metabolic regulation

At the bioenergetic level, AHR exhibits bidirectional control over glycolysis and OXPHOS. In colorectal cancer (CRC), AHR directly interacts with lncRNA-SLCC1 to transcriptionally activate hexokinase 2 (HK2), a key glycolytic enzyme, significantly increasing glycolytic flux and accelerating tumor growth 160. Similarly, separate CRC studies revealed the pivotal glycolytic role of AHR: common APC deficiency activates AHR via the TDO2-Kyn pathway, increasing glycolysis to drive anabolic cancer cell proliferation while promoting CXCL5-mediated macrophage recruitment, which establishes an immunosuppressive microenvironment 161. Conversely, epidermal keratinocyte studies have demonstrated AHR-mediated glycolytic suppression; ChIP-seq analyses have confirmed that AHR binds to promoter regions of the glucose transporter *SLC2A1* and the glycolytic enzyme *ENO1*, inhibiting their transcription and significantly reducing glycolytic flux and pyruvate levels 162. In addition to direct regulation, AHR indirectly modulates mitochondrial metabolism through HIF-1. The HIF-1 heterodimer (HIF-1α/HIF-1β) critically enables CSC adaptation to hypoxia by enhancing glycolytic capacity while suppressing OXPHOS. Mechanistically, AHR activation competes with HIF1A for dimerization partner HIF-1β, sequestering HIF-1β and destabilizing the HIF-1 complex. This disruption impairs basal glycolysis in human coronary artery endothelial cells while enhancing fatty acid oxidation as an alternative fuel source 163. AHR activation significantly suppresses the mitochondrial respiratory chain. Murine muscle tissue studies have demonstrated that AHR activation markedly increases pyruvate dehydrogenase kinase 4 expression and pyruvate dehydrogenase phosphorylation, thereby inhibiting mitochondrial bioenergetic utilization of carbohydrates 164. Exposure to classic AHR activators—Polycyclic aromatic hydrocarbons and their chlorinated derivatives—induces substantial metabolic perturbations; these compounds activate AHR to repress 20 genes associated with the mitochondrial electron transport chain (ETC) while directly interacting with mitochondrial proteins to inhibit complex V and complex I activities 165.

#### AHR and Mitochondrial Quality Control Systems

Multiple studies have indicated that AHR critically regulates mitochondrial quality control. For instance, in oxidative stress models such as H₂O₂-treated melanocytes, AHR potentially protects against oxidative damage by modulating nuclear respiratory factor 1 and its downstream targets to promote mitochondrial DNA synthesis and ATP production. Conversely, impaired AHR activation may compromise mitochondrial repair mechanisms, exacerbating oxidative stress-induced melanocyte apoptosis 166. Similarly, following TCDD exposure in CD4⁺ T cells, early-phase AHR activation alters mitochondrial dynamics: fission gene expression increases, while fusion-related gene expression decreases, concomitant with metabolic reprogramming, as evidenced by significantly reduced cellular respiration and glycolytic rates. Collectively, these findings suggest that AHR impairs cellular metabolism by disrupting quality control mechanisms, thereby compromising metabolic signatures essential for CD4⁺ T-cell activation and differentiation 167. And AHR can also regulates mitophagy. Upon exposure to the toxicant BaP, mitophagy is increased in HaCaT cells to eliminate damaged mitochondria. AHR knockout suppresses BaP-induced mitophagy, restoring the mitochondrial membrane potential (MMP) and ATP levels 168. Moreover, activation activation of AHR by endogenous agonists significantly increases the transcription and protein levels of the mitophagy receptor Bnip3 in hepatocytes. ChIP-seq and luciferase reporter assays demonstrated that AHR interacts with the Bnip3 enhancer region (-10 kb), directly regulating mitophagy 169.

Sustained AHR activation has been shown to induce mitochondrial dysfunction across multiple tissues. In the liver, the potent AHR activator TCDD triggers oxidative stress, partly by disrupting mitochondrial metabolism, leading to inner membrane hyperpolarization, followed by reduced cellular respiration and ATP production. In contrast, AHR-knockout mice exposed to TCDD exhibited significantly lower hepatic ROS generation. Furthermore, PM2.5 induces mitochondrial dysfunction through AHR-mediated CYP1A1 overexpression, resulting in mitochondrial ROS accumulation, mitochondrial permeability transition pore (mPTP) opening, MMP collapse, decreased ATP levels, and downregulation of mRNAs encoding mitochondrial proteins, ultimately impairing cardiomyocyte development in zebrafish embryos. These defects are alleviated by pharmacological or genetic inhibition of AHR 166.

AHR not only localizes to the nucleus to exert canonical transcriptional regulation but also significantly colocalizes with the mitochondrial matrix and outer membrane, suggesting its direct involvement in mitochondrial quality control. Moreover, AHR modulates mitochondrial homeostasis through multiple mechanisms. Notably, although the multifaceted regulation of mitochondria by AHR has been validated in nonneoplastic diseases and diverse tumor entities, its specific role in CSCs remains a critical knowledge gap. For example, the spatial coordination between CSC-specific metabolic plasticity and AHR signaling remains unclear. Deciphering the AHR‒mitochondrion axis in CSCs may provide a theoretical basis for metabolically targeting CSCs and opening new avenues to overcome chemoresistance.

### AHR and epigenetic regulation

**Figure 3:**
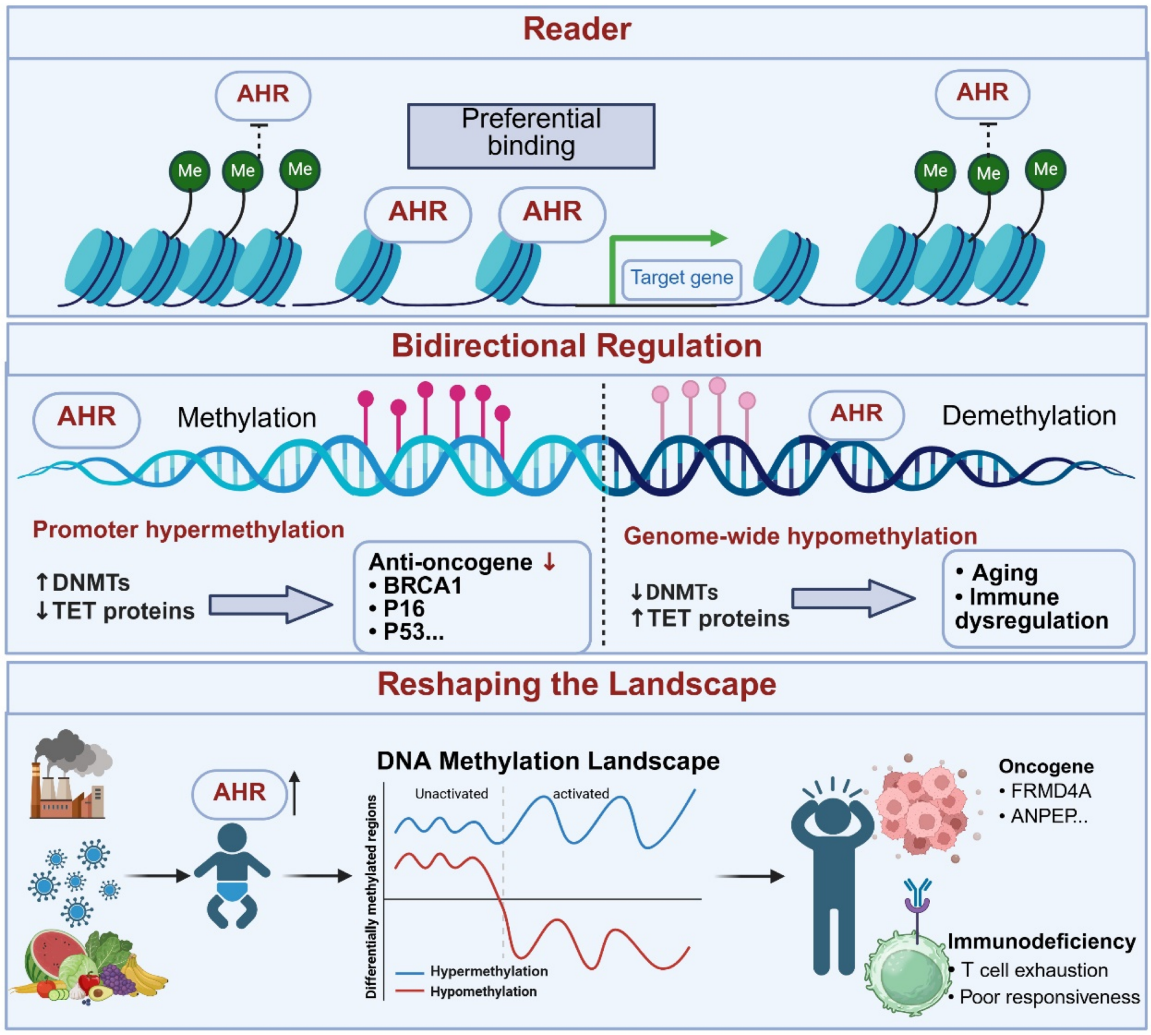
**Role of AHR in DNA Methylation.** AHR acts as a reader of DNA methylation status, preferentially binding to genes in a hypomethylated state. It bidirectionally regulates methylation processes, promoting hypermethylation of tumor suppressor gene promoters to silence their expression and facilitate cancer progression while also positively modulating demethylation. Further studies indicate that AHR activation exerts global rather than localized effects on methylation, leading to sustained and increased variation in methylation patterns. Early-life AHR activation is associated with adult cancer susceptibility and immune alterations. This image was created using BioRender.com.

Epigenetic regulation—As a heritable gene expression regulatory system that is independent of DNA sequence alterations, this mechanism plays a pivotal role as a critical molecular bridge in organisms' responses to environmental stimuli owing to its dynamic reversibility. Its core mechanisms include DNA methylations, posttranslational histone modifications, three-dimensional chromatin structural remodeling, and noncoding RNA regulatory networks. These processes precisely control spatiotemporal gene expression patterns, thereby determining cell fate decisions and maintaining lineage-specific characteristics 170, 171. Recent studies have revealed that tumor heterogeneity substantially originates from the unique phenotypic plasticity of CSCs. These cells exhibit dynamic interconversion between stem-like and differentiated states, leveraging epigenetic reprogramming to evade apoptosis and initiate metastatic progression 151, 172, 173. Notably, AHR, an evolutionarily conserved environmental chemical sensor, plays a pivotal role in maintaining CSC stemness and regulating plasticity. Through ligand-dependent epigenetic regulatory mechanisms, AHR integrates exogenous stimuli with endogenous signaling pathways, offering novel molecular insights into environment‒genome interactions (Figure 3).

#### AHR and DNA methylation

DNA methylation occurs through the addition of a methyl group to the cytosine base at the C5 position, which is catalyzed by DNA methyltransferases (DNMTs), leading to transcriptional activation or suppression of various genes and the regulation of diverse cellular functions—a process tightly controlled by enzymatic reactions 174. Aberrant genome-wide hypomethylation and locus-specific hypermethylation have been reported in multiple tumor tissues, including breast, liver, and ovarian cancers, and serve as hallmarks of cancer development and progression 175-177. The transformation, differentiation, and dedifferentiation of CSCs require precise methylation regulation. Dynamic DNA methylation aberrations, such as promoter hypermethylation of tumor suppressor genes and hypomethylation of proto-oncogenes, disrupt normal cellular processes, thereby promoting carcinogenesis. Furthermore, abnormal DNA methylation interferes with pluripotency-associated transcription and signaling in stem cells, destabilizes the balance between self-renewal and differentiation, and drives the conversion of normal stem cells into CSCs 178-180.

Emerging evidence from epigenetics has shed new light on the tissue-specific regulatory mechanisms of the AHR signaling pathway. Stueve *et al*. (2017) performed whole-genome methylation sequencing of human lung tissues and identified seven hypomethylated regions significantly associated with smoking, including the XRE core binding sites of the canonical AHR target gene *CYP1B1* and its negative feedback regulator AHRR 181. Further mechanistic insight was provided by Miura *et al*. (2021) using HepG2 cell models: β-naphthoflavone (βNF)-activated AHR specifically bound to unmethylated XRE sequences, and the AHR-bound XRE sites remained hypomethylated even when adjacent CpG sites were methylated 182. This finding suggests that chronic AHR ligand exposure may dynamically reshape the epigenome, establishing a positive feedback loop characterized by “AHR activation-hypomethylation-target gene transcription”. Habano *et al*. (2022) proposed that AHR may function as an epigenetic “reader”: its PAS-B domain recognizes the methylation status of XRE sites, preferentially binding to hypomethylated XREs to initiate transcription, whereas hypermethylation causes steric hindrance that disrupts AHR-DNA interactions 183. This mechanism helps explain the functional heterogeneity of AHR across tissues (e.g., liver vs. lung) and tumors (e.g., lung adenocarcinoma vs. hepatocellular carcinoma). Regional methylation patterns may redirect AHR signaling output, thereby influencing processes such as xenobiotic metabolism and immune responses.

The AHR pathway also bidirectionally regulates DNA methylation homeostasis, remodeling the tumor epigenomic landscape. In Triple-negative breast cancer, high AHR expression is correlated with BRCA1 promoter hypermethylation. AHR recruits DNMT1 to the BRCA1 promoter, facilitating CpG island methylation and increasing the expression of proliferation markers (e.g., Cdk4 and Ccnd1). Silencing AHR reversed this repression. Constitutive AHR expression coupled with BRCA-1 promoter CpG hypermethylation may serve as a predictive biomarker for the development of ERα-negative breast tumors 16, 184. Similarly, TCDD induces promoter hypermethylation of p16INK4a and p53 via an AHR-dependent mechanism, inhibiting senescence and promoting aberrant proliferation in keratinocytes 185. Additionally, AHR participates in demethylation processes. PM2.5 exposure disrupts the methylation-demethylation balance through the AHR-ROS axis, resulting in decreased DNMT1/3A expression and increased TET1/3 activity, ultimately accelerating keratinocyte senescence 186. In systemic lupus erythematosus (SLE), the Trp metabolite Kyn suppresses adenosine production in Treg cells via the AHR-TET2 axis, wherein AHR directly binds to the TET2 promoter and enhances its transcription 187.

Recent studies have indicated that AHR-mediated epigenetic changes are not limited to unidirectional hypomethylation or hypermethylation but may comprehensively alter methylation patterns. A population-based study revealed that early-life AHR ligand exposure led to persistent changes in whole-blood DNA methylation patterns that persisted into adulthood. The methylation levels of 11 genes (including cancer-related genes such as FRMD4A [188, 189]and ANPEP 190) correlated with maternal exposure levels. Whole-genome bisulfite sequencing further confirmed that developmental TCDD exposure durably reprogrammed the genome-wide methylation landscape in CD4⁺ T cells, impairing their expansion and effector function in response to influenza virus infection in adulthood. These changes were widespread and complex, involving multiple hyper and hypomethylated regions, thereby promoting polarization of the methylation landscape. Notably, demethylating agents only partially restore CD4⁺ T-cell function 191. In summary, AHR ligands are widespread environmental factors that exert broad, persistent, and multidimensional effects on DNA methylation, profoundly influencing gene expression and cellular function. Further investigations into the role of AHR in epigenetic regulation are crucial for understanding its pathophysiological mechanisms.

#### AHR and Histone Modifications

Histone modifications, as reversible covalent posttranslational protein modifications (e.g., acetylation, methylation, lactylation), regulate gene expression programs by altering the spatial conformation of chromatin 192. In CSCs, these modifications modulate key signaling pathways (WNT, NOTCH, JAK/STAT, etc.), forming a bidirectional regulatory network that synergistically promotes stemness maintenance, self-renewal, and EMT processes. HDACs are closely associated with epigenetic gene silencing and condensed chromatin states in cancer. Overexpression of HDAC has been observed in various cancers, including gastric, prostate, colorectal, esophageal, lung, and breast cancers. On the other hand, multiple studies have reported that HDAC inhibitors are promising therapeutic agents for cancer control 193. Additionally, acetylations can interact with key signaling pathways in CSCs. In CRC,the acetylation level of the Wnt/β-catenin signaling pathway is associated with the homeostasis of colorectal cancer stem cells 194. Furthermore, in multiple myeloma, aberrant activation of the NOTCH ligand JAG2 is closely linked to its acetylation level: loss of function in the nuclear corepressor complex SMRT-HDAC3 elevates histone H3K27 acetylation (H3K27ac) at the JAG2 promoter region, increasing chromatin accessibility and driving JAG2 overexpression 195. Histone methylation influences CSC fate by modulating the activity of pioneer transcription factors. The interaction between the acidic domain of OCT4 and the H3 tail region is regulated by the H3K27 methylation state, which alters nucleosomal DNA exposure sites and modulates the cooperative binding efficiency of downstream factors such as SOX2 196. The H3K4 methyltransferase SETD1A directly sustains the OCT4/SOX2 core regulatory network, and its upregulation is significantly associated with chemotherapy resistance. Preclinical studies have confirmed that SETD1A knockdown effectively suppresses the growth of tamoxifen-resistant cells and CSCs 197, 198.

Histone acetylation positively regulates AHR expression by increasing promoter accessibility. Ding *et al*. (2018) reported that chronic ultraviolet irradiation induces hyperacetylation of histone H3 in skin cells and significantly increases H3K27ac at the AHR promoter region, thereby promoting AHR mRNA expression, activating the AHR-MMP pathway, and ultimately leading to collagen degradation 199. On the other hand, AHR can reciprocally regulate histone acetylation. For example, stimulation with cinnamic acid (CA), an AHR-specific agonist, promotes the formation of an AHR-SRC1 complex that is enriched at the Stc2 gene promoter. This enhances H4K16ac levels, activates Stc2 transcription, and facilitates the repair of chemical-induced damage in hepatocytes 200. In gastric cancer, AHR expression is positively correlated with histone acetylation levels. AHR knockdown significantly inhibits Aza-PBHA-induced histone acetylation and affects cell cycle progression 201. However, in Hepatocellular carcinoma (HCC), AHR expression is positively associated with HDAC levels. AHR directly binds to the HDAC8 promoter to promote its transcription; upregulation of HDAC8 targets the promoter of the tumor suppressor RB1, inhibits its expression, and thereby promotes HCC progression 202, 203.

Histone methylation modulates gene expression and activity, a regulatory mechanism that also extends to the AHR signaling pathway. In a liver cancer model, the histone demethylase LSD1 catalyzes the demethylation of H3K4me3, thereby inducing a repressive chromatin state at the AHR promoter. This erasure of H3K4me3 by LSD1 leads to CpG island hypermethylation and transcriptional silencing of AHR. Treatment with the LSD1 inhibitor SP2509 reverses this epigenetic suppression and restores both AHR mRNA and protein levels 204. Similarly, in a female fibrosis mouse model, KDM5B/KDM5C-mediated modulation of H3K4me3 facilitates coordinated activation of the AHR, Arnt, and Aip genes through chromatin opening remodeling 205. Conversely, AHR can also regulate histone methylation levels. In colitis, the AHR/glycolysis/SIRT1 pathway indirectly modulates H3K9me3 modification in CD4⁺ T cells, effectively reducing H3K9me3 enrichment at the Foxp3 promoter region (positions -1,201 to -1,500), thereby promoting Treg differentiation and alleviating disease progression 206.

#### AHR and non-coding RNAs

The interplay between AHR and non-coding RNAs in epigenetic regulation remains largely unexplored; however, emerging evidence suggests significant research potential in this area. Alluli *et al*. (2023) observed that AHR modulates the expression of multiple lncRNAs, with numerous differentially expressed lncRNAs identified in A549 cells exposed to benzo[a]pyrene (B[a]P) 207. In pancreatic cancer research, environmental toxicant TCDD-activated AHR induces lncRNA MALAT1 expression. MALAT1 subsequently recruits EZH2, the catalytic subunit of PRC2, to deposit H3K27me3 at tumor suppressor gene promoters, thereby mediating epigenetic silencing and promoting cancer progression. This AHR-MALAT1-EZH2 signaling axis mechanistically links environmental exposure to epigenetic dysregulation 208. AHR also participates in epigenetic pathways governing homologous recombination (HR) repair in recurrent miscarriage. As a transcription factor, AHR promotes lnc-HZ10 transcription. Conversely, lnc-HZ10 upregulates AHR expression by inhibiting CULB4-mediated ubiquitination and degradation, establishing a positive feedback loop. This lnc-HZ10/AHR axis impairs HR repair of double-strand breaks (DSBs) in human trophoblast cells by reducing BRCA1 nuclear abundance and compromising BRCA1 recruitment to DSB foci, ultimately leading to DSB repair defects 209.

## AHR and Cell Fate: Modes of Cell Death

**Figure 4:**
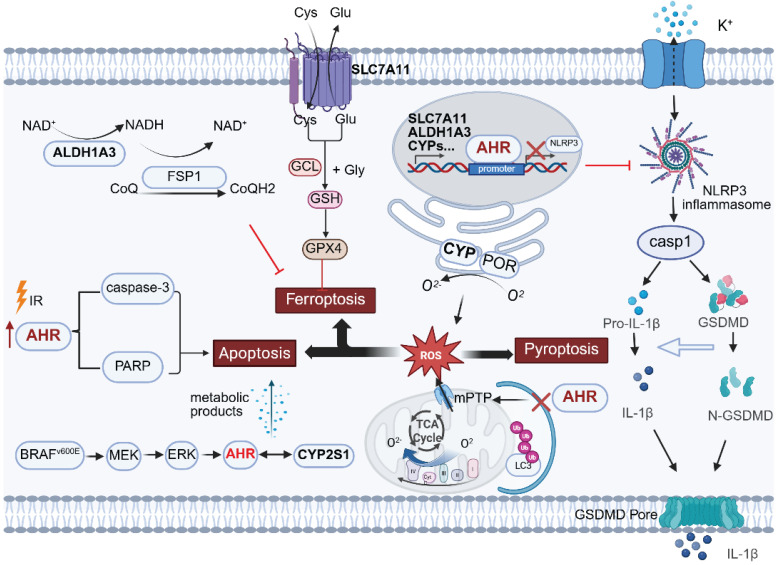
**Regulatory role of AHR in programmed cell death.** AHR differentially modulates ferroptosis, apoptosis, and pyroptosis across cell types. The AHR-CYP-ROS signaling axis represents a common pathway in these processes. Transcriptional activation of CYP enzymes by AHR leads to O^2-^ release during catalytic cycles, inducing oxidative stress imbalance and triggering cell death execution programs. This image was created using BioRender.com.

The goal of cancer therapy is to eliminate tumor cells while minimizing damage to normal cells. However, due to tumor resistance, cancer treatment remains challenging. Thus, investigating the signaling pathways and molecular mechanisms underlying distinct cell death modalities may facilitate the discovery of novel antitumor strategies or the optimization of existing therapeutic regimens (Figure 4).

### Ferroptosis

Ferroptosis is an iron-dependent regulated cell death driven by oxidative damage and membrane disruption 210. It interacts with tumor suppressors such as TP53 211, 212 and KEAP1 213, 214 to exert its antitumor effects. Owing to metabolic reprogramming, genetic mutations, and imbalanced ferroptosis defenses, certain cancers, including breast cancer and small cell lung cancer, exhibit exceptional susceptibility to ferroptosis 215-218. Ferroptosis-targeting interventions—including agents like IFN-γ, sorafenib, and sulfasalazine, as well as ferroptosis-immunotherapy combinations— effectively suppress tumor progression and overcome drug resistance 211, 219, 220. Zhang *et al*. (2024) found that the gut microbiota-derived metabolite trans-3-indoleacrylic acid (IDA) confers ferroptosis resistance in CRC. IDA activates AHR as an exogenous ligand, upregulating ALDH1A3 and AIFM2 (FSP1) to enhance NADH regeneration and maintain redox homeostasis. AHR knockout reverses IDA-induced tumor growth and restores ferroptosis susceptibility 221. The regulatory role of AHR in ferroptosis extends to NSCLC: studies have confirmed AHR directly binds the SLC7A11 promoter to increase its expression, enhancing glutathione synthesis and ROS scavenging, thereby inhibiting ferroptosis and promoting cancer progression 222.

Conversely, in intestinal intraepithelial lymphocytes (IELs), AHR hyperactivation promotes ferroptosis: loss of AHRR sustains AHR signaling, inducing *CYP1A1* expression. *CYP1A1* generates superoxide anions via electron leakage, triggering lipid peroxidation. Consequently, AHRR-/- mice exhibit broad IEL deficiencies, which are reversed by dietary selenium or vitamin E supplementation 223. Thus, AHR signaling must be tightly regulated to prevent oxidative stress imbalance and uncontrolled ferroptosis.

### Apoptosis

Apoptosis is an energy-dependent programmed cell death process that eliminates dysfunctional or abnormal cells in response to stressors such as hypoxia, DNA damage, or viral infection 224, 225. It involves caspase cascade activation or mitochondrial cytochrome C release and has been extensively studied as a cancer therapeutic target 226.

AHR serves as a key sensor for environmental chemicals, with its role in apoptosis varying by external signals. The canonical AHR/ARNT complex transcriptionally activates CYPs to regulate redox homeostasis. During catalytic reactions, CYPs generate sustained ROS via “uncoupling cycles,” increasing proapoptotic stress 227. In an HCC model, Zhan *et al*. (2022) demonstrated that ionizing radiation (IR) significantly elevated cleaved PARP and cleaved caspase-3 levels by inducing AHR overexpression, promoting tumor cell apoptosis. Conversely, AHR degradation reduces ROS production and reverses IR-induced apoptosis, revealing the proapoptotic function of the AHR-CYP axis in oxidative stress responses 228. Conversely, in uterine leiomyoma, the phthalate metabolite MEHHP activates AHR via the Kyn pathway, suppressing apoptosis; AHR knockout or CH223191 abolishes this survival effect 229. In BRAF V600E-mutant thyroid cancer, MAPK/ERK signaling upregulates AHR, which induces CYP2S1. CYP2S1-generated metabolites act as endogenous AHR ligands, creating an autocrine loop that drives proliferation, invasion, and apoptosis resistance 230.

### Pyroptosis

Pyroptosis is gasdermin-mediated programmed necrosis marked by pore formation, osmotic lysis, and release of DAMPs and IL-1β/IL-18 231. Localized tumor pyroptosis remodels the immune microenvironment and synergizes with immune checkpoint inhibitors to activate antitumor immunity 232. In renal injury models (BDE-47/Cd), AHR pathway activation correlates with tubular epithelial pyroptosis; the AHR antagonist CH223191 reduces cleaved caspase-3 and GSDME-NT, confirming AHR involvement 233. In BaP-induced myocardial infarction, AHR suppresses PINK1/Parkin mitophagy, triggering mPTP overactivation, ROS release, and NLRP3 inflammasome activation, forming a pyroptotic feedback loop 234. These findings suggest tissue-specific regulatory mechanisms of AHR in different injury models. Conversely, AHR negatively regulates NLRP3 in macrophages: AHR binds the NLRP3 promoter XRE to suppress transcription, reducing caspase-1 activation and IL-1β secretion 235. In a colitis model, myeloid-specific AHR deficiency exacerbates macrophage pyroptosis, whereas supplementation with an AHR ligand precursor inhibits potassium efflux and NLRP3 assembly via Odc1-driven polyamine biosynthesis, thereby suppressing pyroptosis and alleviating colitis symptoms 236.

Notably, while the mechanistic link between AHR and pyroptosis has been extensively studied in inflammatory diseases, their interplay within the tumor microenvironment remains a significant knowledge gap requiring further investigation.

### AHR signaling exhibits dual tumor immunomodulatory effects

**Figure 5:**
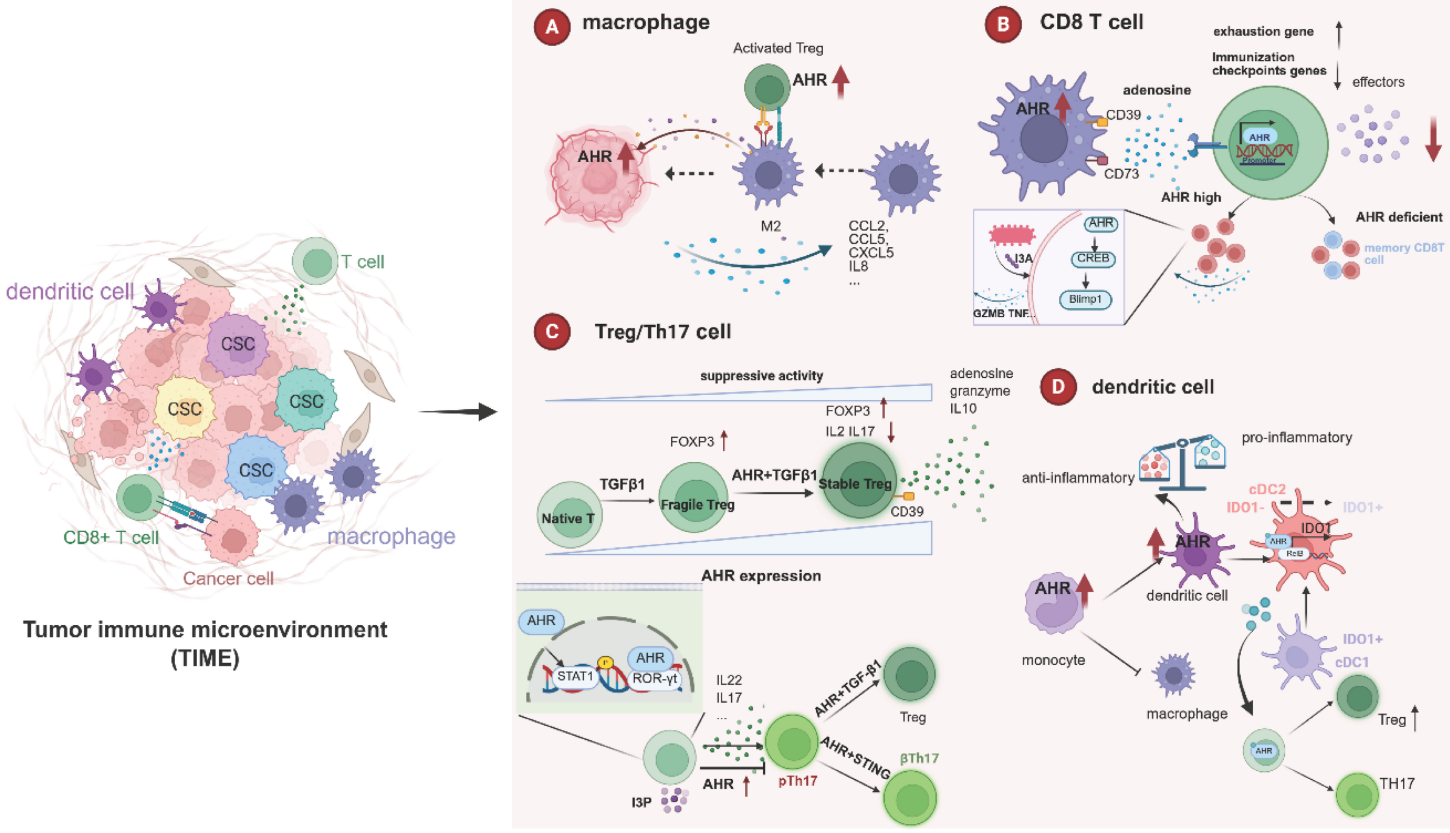
**Role of AHR in the tumor immune microenvironment.** AHR plays a multifaceted role within the tumor immune microenvironment (TIME), exerting distinct effects on various immune cell components. AHR regulates the recruitment of TAMs and drives their polarization toward an immunosuppressive phenotype. It further enhances the immunosuppressive function of macrophages via the Treg‒macrophage interaction axis. In CD8⁺ T cells, AHR occupies a central position in the exhaustion program: AHR signaling derived from TAMs drives CD39 expression and collaborates with CD73 to catalyze adenosine production. This promotes significant upregulation of exhaustion markers (e.g., Pdcd1, Gata3, Ikzf2) and suppresses effector molecule production in CD8⁺ T cells. Moreover, AHR is crucial for the development of highly activated and polyfunctional tumor-infiltrating CD8⁺ T cells (CD8 TILs); AHR-deficient CD8⁺ T cells exhibit reduced polyfunctional and activated (e.g., Ifit1^+^, Il1b^+^) subsets but increased populations with a central memory-like phenotype. AHR is also involved in Treg development, where its expression level is closely correlated with Treg survival and suppressive activity. AHR governs the differentiation of Th17 cells through multiple mechanisms, including the STAT1-RORγt pathway, which regulates key cytokines such as IL-17 and IL-22. Critically, AHR is essential for the conversion of Th17 cells into Treg cells, a process reduced by AHR antagonists. AHR acts as a molecular switch for monocyte fate: its activation promotes monocyte-derived dendritic cell (mo-DC) differentiation via BLIMP-1 induction while suppressing the proinflammatory phenotype of DCs. AHR also participates in DC-mediated T-cell activation and differentiation. Importantly, the acquisition of IDO1-dependent tolerogenic activity by the cDC2 subset requires AHR expression. This image was created using BioRender.com.

AHR has emerged as a pivotal regulator within the tumor immune microenvironment. By responding to endogenous ligands such as Trp metabolites (e.g., Kyn), AHR orchestrates immunosuppressive networks through modulating tumor-associated macrophage (TAM) polarization, regulatory T-cell (Treg) differentiation, and effector T-cell exhaustion (Figure 5). Notably, crosstalk between AHR signaling and immune checkpoints (e.g., PD-1/PD-L1) directly influences checkpoint molecule expression, thereby impacting immunotherapy efficacy. Targeting AHR pathways to counteract tumor immune evasion represents a promising frontier in cancer immunotherapy.

#### AHR and T cells

T cells play a pivotal role in maintaining human health and combating disease, and they serve as a cornerstone of cancer immunotherapy.CD8⁺ T cells are core antitumor effectors that kill tumor cells via granzyme B/perforin and cytokine secretion (IFN-γ, TNF-α), while also inhibiting angiogenesis and enhancing DC function 237, 238. They generate memory subsets for long-term surveillance 239, 240. thus, durable CD8⁺ T-cell responses directly determine tumor recurrence and patient survival. However, in progressive tumors, chronic antigen stimulation drives CD8⁺ T cells into exhaustion—characterized by sustained inhibitory receptor expression (PD-1, LAG-3, TIM-3) and loss of effector function 237, 241, 242. Reinvigorating exhausted TILs is therefore a key strategy to enhance immunotherapy efficacy. Takenaka *et al*. (2019) linked AHR activation to CD8⁺ T-cell exhaustion in the TME. Tumor-derived Kyn activates AHR in TAMs, inducing CD39 expression, which synergizes with CD73 to produce adenosine. Adenosine-A2AR signaling upregulates exhaustion markers (Pdcd1, Gata3, Ikzf2) and suppresses IFN-γ/TNF-α secretion in CD8⁺ T cells 243. Liu *et al*. (2021) further confirmed AHR's central role in exhaustion: AHR inhibition or knockout reduced inhibitory receptor expression and restored IFN-γ/TNF-α production. Mechanistically, AHR directly binds promoters of exhaustion-related genes (PDCD1, LAG3, HAVCR2, ENTPD1), revealing its critical function as a transcription factor in CD8⁺ T-cell exhaustion 244. Conversely, single-cell analysis revealed AHR is essential for generating highly activated, polyfunctional CD8⁺ TILs. AHR-deficient CD8⁺ T cells showed reduced polyfunctional and activated subsets but increased circulating memory-like phenotypes, leading to accelerated tumor growth 245. Notably, AHR-mediated regulation of CD8⁺ T cells involve the microbiota-metabolite axis. In a melanoma model, the tryptophan metabolite indole-3-aldehyde (I3A) derived from *Lactobacillus reuteri* (Lr)activates AHR signaling in CD8⁺ T cells via the AHR-CREB-Blimp1 axis, thereby promoting an immunostimulatory phenotype 246.

The delicate balance between Th17 cells and regulatory T (Treg) cells is critical in the pathogenesis of inflammatory diseases and tumors 247, 248. In various cancer types, extensive Treg infiltration within the tumor microenvironment is commonly observed and is closely associated with advanced disease stages, the presence of metastases, and poor prognosis 249-251. As key mediators of tumor immune evasion, Treg cells impede effective antitumor immunity by suppressing immune responses and preventing the recognition and elimination of tumor cells 252, 253. Th17 cells exert dual effects on tumor progression depending on the tumor context. In hepatocellular carcinoma, Th17 cells promote migration and invasion via the TWEAK-Fn14 signaling axis 254. Conversely, in melanoma, tumor-specific Th17 cells demonstrate superior antitumor efficacy compared with other CD4⁺ T-cell subsets, eradicating tumors more effectively and protecting against distant metastasis through distinct host immune regulatory mechanisms 249.

Multiple studies have demonstrated that AHR participates in Treg development. For example, the exogenous administration of synthetic AHR agonists suppresses various autoimmune diseases, including experimental autoimmune encephalomyelitis 255, colitis 256, and collagen-induced arthritis 257. Moreover, approximately 40-50% of AHR knockout mice die shortly after birth due to inflammatory infiltration in multiple organs. These mice exhibit an approximately 30% reduction in Treg cells, with the remaining surviving Tregs showing significantly decreased FOXP3 levels 258. Further supporting this, Griffith *et al*. (2025) demonstrated that AHR activation within CD4⁺ T cells can trigger Treg differentiation. Analysis of human pancreatic tissues revealed that smokers harbor markedly higher AhR activity than non-smokers, accompanied by a pronounced enrichment of Tregs in the tumor microenvironment. Subsequent experiments demonstrated that AhR signaling in CD4⁺ T cells orchestrate the co-accumulation of both Treg and TH22 cells, thereby attenuating CD8⁺ T-cell effector function and ultimately promoting pancreatic tumorigenesis and progression 259. Similarly, Ye *et al*. (2017) employed gene-editing technology to label AHR in murine Treg cells, revealing that, compared with other peripheral organs, AHR is preferentially expressed by microbiota-independent intestinal Tregs. In a T-cell transfer model of colitis, AHR-expressing Tregs demonstrated greater *in vivo* suppressive activity than AHR-deficient Tregs did 260. Importantly, TGF-β, a crucial cytokine influencing Treg differentiation both *in vivo* and *in vitro*, has synergistic effects with AHR. Notably, naïve human CD4^+^ T cells activated by TGF-β expression do not necessarily display suppressive activity. However, when AHRs are simultaneously active, they indeed acquire functional Treg characteristics, including high FOXP3 expression; low expression of IFNA1, IL2, and IL17; and CD39-dependent suppressive responsiveness 261.

Th17 cells are a distinct CD4⁺ T-cell subset defined by RORγt expression and production of IL-17/IL-22, playing critical roles in inflammatory diseases 262, 263. AHR regulates Th17 cell differentiation through multiple mechanisms. AHR directly modulates IL-17 expression by binding DRE sites in the Th17 promoter 264. It physically interacts with ROR-γt, as demonstrated by PLA assays in SLE and RA patient T cells 265 and cooperates with c-Maf to promote IL-22 production 143. However, AHR effects are context-dependent: linoleic acid acts as an AHR antagonist under Th17-polarizing conditions, driving Signal transducer and activator of transcription 1 (STAT1) phosphorylation (Ser727) to suppress IL-17 while increasing Foxp3, thereby disrupting Th17/Treg balance and exacerbating colitis 266. Conversely, in SLE mice, MSC-derived indole 3 pyruvate (I3P) activates AHR to suppress Th17 cells (IL-17A⁺ cells reduced from 47.3% to 25.7%) and delay disease progression 267. Furthermore, the presence of AHR is critical for Th17 cell plasticity. Intestinal Th17 cells can transdifferentiate into Treg cells under the synergistic influence of TGF-β1 (via Smad3 signaling) and AHR activation. Supplementation with the AHR ligand FICZ significantly enhances the generation of Tr1exTh17 cells, whereas AHR antagonism suppresses this conversion. These *in vitro*-generated Tr1exTh17 cells exhibit regulatory function 268. Furthermore, Damasceno *et al*. (2022) demonstrated that STING-induced conversion of pathogenic Th17 cells to non-pathogenic Th17 cells requires AHR signaling to induce IL-10 production while suppressing IL-17A and IL-23R expression 269.

#### AHR and macrophages

As key members of the mononuclear phagocyte system, macrophages maintain tissue homeostasis through immune regulation, phagocytosis, and antigen presentation 270. In the TME, tumor-associated macrophages (TAMs) represent the most abundant immune cell population and are broadly categorized into classically activated M1-like and alternatively activated M2-like subtypes. M1 macrophages are generally considered tumoricidal, whereas M2 macrophages exert immunosuppressive functions and promote tissue repair as well as tumor progression. Both M1- and M2-like TAMs coexist throughout all stages of tumor development, with the proportion of M2-like TAMs increasing as the disease progresses 271, 272. Meta-analyses across multiple cancer types (cumulative N > 10,000) have confirmed that high TAMs density correlates with reduced recurrence-free and overall survival 273-275.

AHR regulates multiple aspects of macrophage recruitment to the TME. In APC-deficient CRC, Tryptophan 2,3-dioxygenase (TDO2) metabolizes tryptophan through the KP, thereby activating AHR. The TDO2-Kyn-AHR axis promotes tumor progression through dual mechanisms: enhancing cancer cell glycolysis and upregulating CXCL5 to recruit CCR2⁺ TAMs into the tumor core 161. In BRCA1-associated breast cancer models, AHR promotes CD14⁺ monocyte recruitment by regulating CCL2/CCL5 secretion; these monocytes differentiate into proangiogenic TAMs that facilitate neovascularization via VEGF-A/PDGF-BB 276. AHR also regulates TAM polarization. In PDAC, TAMs show upregulated AHR and CYP1B1. Macrophage-specific AHR knockout reverses immunosuppressive phenotypes, enhancing M1 polarization (increased MHC-II, CD40, PD-L1) and reducing proliferation while increasing apoptosis 13. Additionally, single-cell transcriptomic analysis of glioblastoma revealed that macrophage subsets with elevated AHR activity display prominent M2 characteristics, with minimal enrichment of M1 genes. TCGA database analysis further revealed strong positive correlations between AHR pathway genes (IDO1/TDO2/CYP1B1) and M2 markers (CD206, CD14), Treg-related genes (FOXP3, IL2RA), and immune checkpoints (PD-1, CTLA-4) 277. Current evidence suggests that AHR drives the immunosuppressive polarization of TAMs.

In addition to directly inducing the immunosuppressive polarization of TAMs, AHR can also interact with other cells to reinforce the TAM-regulated immunosuppressive microenvironment. On the one hand, First, via the Treg-macrophage axis, Kyn-pretreated Tregs drive M2-like macrophage polarization through AHR signaling. In coculture systems, Kyn increased the proportion of CD206⁺ M2-like macrophages from 20% to 40% (p<0.05), an effect reversed by the AHR inhibitor CH-223191. Moreover, Kyn gradients correlated with BM-APC-mediated CD8⁺ T-cell suppression in wild-type but not AHR-knockout Tregs, indicating AHR dependence 278. On the other hand, AHR regulates the immune checkpoint CD155 on TAMs. *In vivo* AHR inhibition reduced both TAM density and CD155 expression on TAMs, relieving competitive binding to the T-cell receptor CD226 and reversing immunosuppression 279.

#### AHR and DC

Dendritic cells (DC), the most potent professional antigen-presenting cells (APCs) in the immune system, efficiently capture, process, and present antigens. They play pivotal roles in initiating, regulating, and sustaining immune responses by inducing potent, antigen-specific T-cell immunity, positioning them at the center of immune response orchestration.

AHR serves as a molecular switch for monocyte fate determination 280, 281. In *in vitro* culture models, AHR activation promotes monocyte-derived DC (mo-DC) differentiation through BLIMP-1 induction while impairing monocyte-to-macrophage differentiation. AHR deficiency also compromises mo-DC differentiation *in vivo*. Mice fed an I3C-supplemented diet presented significant increases in cutaneous mo-DC with concomitant reductions in MHC class II^+^ macrophages 282. AHR suppresses proinflammatory phenotypes in DCs. Nguyen *et al*. (2010) demonstrated that LPS/CpG stimulation markedly upregulates AHR expression in bone marrow-derived DCs. Genetic ablation of AHR significantly attenuated IL-10 secretion upon TLR activation, suggesting that AHR establishes a negative feedback loop to constrain excessive inflammatory responses 283. Platzer *et al*. (2009) revealed that the AHR agonist VAF347 inhibits the immunocompetence of human mo-DC through two mechanisms: reducing IL-12/IL-23 secretion upon anti-CD40/TNF-α stimulation and impairing the differentiation efficiency of CD34^+^ hematopoietic precursors into DCs and Langerhans cells 284.

AHR participates in DC-mediated T-cell activation and differentiation. AHR activation in DCs induces IDO1/IDO2 expression, increasing Kyn production. Kyn promotes Treg differentiation while suppressing Th17 polarization through AHR-dependent mechanisms, establishing an immune-tolerant microenvironment 285. Moreover, AHR plays an essential role in IDO1-tolerogenic pathway acquisition by DC subsets. The tolerogenic IDO1 pathway is expressed in mature cDC1s but absent in cDC2s. The activated IDO1 enzyme in cDC1s releases the Trp metabolite L-Kyn, which acts in a paracrine manner to propagate tolerogenic activity from the cDC1 to the cDC2 subsets. This process requires AHR and its coactivator RelB to regulate IDO1 expression in cDC2s, thereby eliciting IDO1-dependent tolerogenic activity 286.

#### AHR and immune checkpoints

Upregulation of immune checkpoint expression has been demonstrated to promote tumor immune escape. Immune checkpoint inhibitor therapy, which revitalizes antitumor immunity, constitutes a core component of cancer treatment and has shown unprecedented efficacy in a subset of cancer patients.

AHR directly regulates the expression of the immune checkpoint PD-L1. In breast cancer, elevated expression of AHR and PD-L1 correlates with increased Treg infiltration and poor prognosis. Mechanistically, GM-CSF secreted by breast cancer cells upregulates AHR expression in macrophages within the premetastatic niche (PMN). Activated AHR then binds to the *Pdl1* promoter, inducing PD-L1 expression, which in turn promotes Treg differentiation and establishes an immunosuppressive PMN 287. Further mechanistic insights come from Wang *et al*. (2021), whose analysis of the PD-L1 promoter revealed five potential consensus AhR response elements (AHREs). Mutations in these predicted AHREs significantly impaired AHR-mediated regulation of PD-L1, demonstrating that AhR directly transactivates the PD-L1 promoter 288. And Han *et al*. (2023) analyzed 168 patient samples and found that the combination of nuclear AHR and membrane PD-L1 outperformed membrane PD-L1 alone by 29.7% in predicting patient survival benefit, providing a simple and effective approach for prognostic prediction in immunotherapy 289.

IDO1 has emerged as a prominent immune checkpoint closely associated with AHR signaling. Distinct from classical checkpoints such as PD-1/PD-L1, which mediate direct contact-dependent inhibition, IDO1 primarily functions through metabolic reprogramming of the tumor microenvironment 290-292. The core mechanism of IDO-KYN-AHR pathway involves indoleamine-2,3-dioxygenase-mediated Trp catabolism, generating Kyn, which activates AHR to modulate immune cell function 293, 294. This pathway plays pivotal roles in shaping the tumor immune microenvironment, particularly in Treg differentiation and effector T-cell (e.g., CD8^+^ T cell) suppression, which are closely associated with tumor immune evasion and tolerance 295, 296. In CRC, while tumor cell supernatants had minimal effects on CD4^+^ T cells, they significantly inhibited CD8^+^ T-cell function. Further studies revealed that tumor-derived Kyn activated AHR in CD4^+^ T cells, increasing FoxP3 transcription to promote Treg differentiation. Treg expansion subsequently suppresses CD8^+^ T-cell proliferation and cytotoxicity, ultimately establishing an immunosuppressive microenvironment favorable for tumor progression 297. In atherosclerosis-related diseases, the IDO-KYN-AHR pathway has similar functions. DC-expressed IDO activates AHR in CD4^+^ T cells to promote Treg differentiation, thereby modulating local immune responses 298. In addition, in pancreatic cancer, blocking KP and targeting the Kyn-AHR pathway to blunt the migration and invasion of cancer cells exhibited preclinical efficacy and ameliorated IDO1/TDO-mediated immunosuppression in pancreatic cancer model mice 299. Furthermore, in TRC, IDO-KYN-AHR activation enables escape from IFN-γ-induced apoptosis and induces immunodormancy. Mechanistically, this pathway upregulates the cell cycle inhibitor p27, causing cell cycle arrest and TRC dormancy. Additionally, p27 binds cytoplasmic phosphorylated STAT1 (p-STAT1), inhibiting its nuclear translocation and biasing signaling toward the IDO-AHR cascade 300. Studies have shown that combining IFN-γ with IDO/AHR inhibitors effectively eliminates tumors *in vivo*, providing novel immunotherapeutic strategies.

In addition, AHR can induce IDO expression, with TCDD stimulation increasing IDO1 levels 5.4-fold *in vitro* 301. Moreover, AHR is essential for sustained IDO expression in DCs. AHR-/- mice exhibit complete absence of the IDO2 protein during the early and late DC maturation stages, along with significantly reduced IDO1 in the late stages. AHR-/- DCs showed threefold reduced tolerogenic activity and impaired Treg induction capacity. While exogenous Kyn fully reversed IDO enzymatic inhibition, it failed to restore persistent IDO expression in AHR-/- DCs, confirming the absolute requirement of AHR complex binding for maintaining tolerogenic IDO expression. IDO enables DCs to rapidly switch from immunogenic to tolerogenic phenotypes, mediating robust Treg expansion while blocking IL-12-driven CTL activation, contributing to therapy resistance. Targeting the DC-AHR-IDO axis (e.g., through DC-specific AHR modulators) may represent a novel strategy to overcome tumor immune resistance and benefit cancer patients 302.

IL4I1 has emerged as a novel metabolic immune checkpoint, exhibiting aberrant expression across diverse cancer types. It drives tumor immune evasion, promotes cancer progression, and contributes to immunotherapy resistance through multiple mechanisms: suppressing T-cell proliferation and effector functions, and negatively regulating B-cell receptor and T-cell receptor signaling 303. In exploring alternative pathways of AHR activation, Sadik *et al*. (2020) employed WGCNA analysis across multiple solid tumors and found that IL4I1 exhibits a stronger correlation with AHR activity than other Trp-catabolizing enzymes such as TDO2 and IDO1. Further mechanistic studies in glioblastoma revealed that IL4I1 catalyzes Trp to generate the key intermediate metabolite I3P, which activates AHR to enhance GBM invasiveness while suppressing CD8⁺ T-cell proliferation. Notably, I3P activates AHR at substantially lower concentrations compared with Kyn 304. Similarly, in lung cancer, IL4I1⁺ macrophages promote Trp degradation and AHR activation, driving CD8⁺ T-cell exhaustion and conferring resistance to anti-PD-1 therapy 305. However, despite the ability of IL4I1-derived metabolites to activate AHR, their correlation with AHR target gene expression in cancer remains relatively weak 306. Moreover, the utilization patterns of IL4I1 and the kynurenine pathway appear to differ markedly across cancer types 307. Nonetheless, the central importance of AHR as the ultimate effector integrating multiple Trp metabolic pathways remains unequivocal. Collectively, these findings suggest that IL4I1 may compensate for the limitations of IDO1 inhibitors. However, the relationship between IL4I1 and AHR is far from a simple linear regulatory axis; rather, it is highly context-dependent. A comprehensive understanding of the interplay between IL4I1 and IDO1/TDO2 is essential for achieving precise modulation of AHR signaling and overcoming cancer immunotherapy resistance.

#### AHR and microbiota-metabolite

Recent studies have increasingly focused on the interplay between AHR and the microbiota, particularly within the context of intestinal immunity—a finding consistent with AHR's established role as an environmental sensor 2, 308. The gut microbiota serves as a critical source of AHR ligands: on one hand, it regulates immunity through Trp-derived metabolites that engage the IDO1/TDO2-Kyn-AHR axis; on the other, certain bacteria directly produce high-affinity AHR ligands to activate AHR signaling and sustain intestinal immune homeostasis 27, 309, 310. Notably, analogous regulatory paradigms have recently been identified in the tumor microenvironment, where microbiota-derived metabolites modulate antitumor immunity via AHR. For instance, engineered probiotic bacteria producing indole-3-acetic acid have been shown to activate AHR, thereby reshaping the tumor immune landscape by enhancing T-cell infiltration and cytokine production 311. These findings suggest that supplementing with probiotics or modulating microbial metabolism may represent a promising adjunct strategy for cancer therapy.

AHR orchestrates tumor immunity through the microbiota-metabolite axis. In melanoma, Lr metabolizes Trp to produce I3A, which activates AHR signaling in CD8⁺ T cells. This induces an immunostimulatory phenotype characterized by Tc1 expansion, increased IFN-γ and granzyme B secretion, and downregulation of exhaustion markers. Mechanistically, I3A enhances Blimp1 transcription via the AHR-CREB axis to potentiate cytotoxicity. Notably, the antitumor effects of Lr/I3A require CD8⁺ T-cell-intrinsic AHR activation, as they are abrogated in AHR-deficient CD8⁺ T cells 246. Similarly, *Lactobacillus gallinarum* synergizes with anti-PD1 therapy by reducing intratumoral Treg infiltration and enhancing CD8⁺ T-cell effector function. Its metabolite indole-3-carboxylic acid (ICA) acts through dual mechanisms: inhibiting IDO1 to suppress Kyn production, and competitively binding AHR to block Kyn-induced Treg differentiation. ICA phenocopies the effects of *L.* gallinarum, significantly enhancing anti-PD1 efficacy in CRC—an effect reversed by Kyn supplementation 312. Conversely, certain microbes exploit AHR to promote immunosuppression. In head and neck squamous cell carcinoma (HNSCC), chronic stress-induced oral dysbiosis elevates Kyn levels. Kyn stabilizes AHR via deubiquitination, driving CD8⁺ T-cell exhaustion and accelerating tumorigenesis 313. Likewise, *Fusobacterium nucleatum* and its outer membrane vesicles activate the TDO2/AHR axis in tumor-associated macrophages, upregulating immunosuppressive cytokines and checkpoints while suppressing CD8⁺ T-cell and CTL infiltration and enhancing Treg recruitment 314.

Focusing on probiotic adjunctive therapy or targeting microbial metabolism in tumor immunotherapy may serve as a key strategy to enhance therapeutic efficacy and overcome drug resistance. By intervening in microbial metabolism to reshape AHR signaling, it is possible to achieve a shift in the immune microenvironment from pro-tumor to anti-tumor, offering new insights for improving immunotherapy outcomes.

### AHR-related clinical trials

AHR plays dual roles in cancer progression and immunomodulation. It directly regulates cancer stemness maintenance, mitochondrial metabolic reprogramming, and cell death modalities. Simultaneously, it transcriptionally governs the differentiation and functionality of both innate and adaptive immune cells. Although AHR exhibits cell type-specific heterogeneity in its immunomodulatory effects, its overall activation promotes a broadly immunosuppressive TME. Despite promising preclinical evidence supporting AHR-targeted strategies, their clinical translation faces substantial challenges. As summarized in Table 2, current AHR-related clinical trials in oncology remain limited (e.g., NCT05472506, NCT04999202, and NCT04200963), with most focusing on combination regimens with immune checkpoint inhibitors.

Tapinarof cream, the first therapeutic AHR modulator approved globally, has significant clinical value in treating inflammatory skin diseases, including psoriasis and atopic dermatitis (AD), indicating the successful translation of AHR-targeted therapy. Its tripartite mechanism involves immunomodulation through the suppression of Th17 cytokines, antioxidant activation via Nrf2 pathway enhancement with direct ROS scavenging, and barrier repair via the regulation of structural proteins to inhibit epidermal hyperplasia 315. This nonsteroidal agent overcomes the limitations of conventional glucocorticoid therapies by balancing efficacy with safety, with key clinical trials confirming therapeutic benefits. A phase III psoriasis study revealed that 40.9% of patients achieved complete lesion clearance, and 58.2% attained ≥2-grade Physician Global Assessment improvement after 12 weeks of treatment, maintaining a 122-day median relapse-free interval postcessation 316, whereas an open-label AD extension study reported a 51.9% clearance rate after 48 weeks of therapy, with a 79.8-day sustained efficacy posttreatment 317. Following FDA approval of Dermavant's VTAMA® cream (NDA 215272) for psoriasis in May 2022, Organon received December 2024 expanded approval for AD treatment in patients aged ≥ 2 years, establishing tapinarof as the first pediatric AHR-targeted agent whose durable control potential (> 3-month remission postdiscontinuation).

IK-175, a novel AHR-targeted therapeutic, exerts antitumor effects by remodeling the immunosuppressive TME. This compound directly binds AHR with high affinity, demonstrating potent AHR activity inhibition across species (mice, rats, monkeys, and humans). Preclinical studies have revealed favorable ADME properties and linear pharmacokinetics. Notably, while IK-175 lacks direct antiproliferative effects on human or murine cancer cell lines, it effectively reverses immunosuppression by promoting proinflammatory cytokine release from T cells, suppressing immunosuppressive T-cell differentiation, and converting tumor-draining lymph nodes and intratumoral microenvironments toward proinflammatory states. In combination studies, IK-175 synergizes with anti-PD-1 antibodies or liposomal doxorubicin, significantly enhancing antitumor efficacy, supporting its broad therapeutic potential in combination regimens 10.

Research on the Kyn pathway underscores the therapeutic value of AHR inhibition. BAY 2416964 targets downstream KYN-AHR signaling to overcome resistance to upstream inhibitors. Although IDO1/TDO2 initially attracted significant interest 5, the clinical failure of IDO1 inhibitors reflects mechanistic limitations: they neither block TDO2-mediated KYN production nor counteract immunosuppressive metabolites downstream of IL4I1 5. Targeting the KYN-AHR signaling nexus may offer superior therapeutic advantages. Through high-throughput screening, BAY 2416964 was identified as a novel AHR inhibitor that dose-dependently reverses ligand-induced AHR activation in human and murine immune cells, with excellent pharmacokinetic properties. This compound not only reverses AHR-mediated immunosuppression but also induces the T-cell effector IL-2 more potently than the IDO1 inhibitor epacadostat does. When combined with anti-PD-1, it amplifies T-cell responses, providing a new strategy to overcome KYN pathway-driven immune checkpoint inhibitor resistance 11.

AHR inhibitors have evolved from "environmental toxin receptor antagonists" to "immune microenvironment modulators," with core value in dismantling tumor resistance networks and reprogramming immunosuppressive ecosystems. With the clinical advancement of agents such as IK-175 and BAY 2416964—coupled with the structure-based development of next-generation inhibitors—AHR-targeted therapies are poised to become standard components in solid tumor combination regimens within five years. Future efforts must address tissue-selective delivery and dynamic resistance monitoring to achieve truly personalized intervention.

### AHR-targeting nanoparticles

Nanomaterials, with their unique physicochemical properties, have opened new avenues for precision targeting of the AHR. These nanoparticles can be synthesized from diverse sources—including natural products, metals, and polymers—and functionalized for various medical applications 318-320. Their core advantages include: overcoming the low bioavailability of conventional drugs, and addressing poor tumor specificity through passive targeting (enhanced permeability and retention effect) and active modification, thereby enabling precision, high-efficacy, and multifunctional therapy 321-323. Currently, AHR-targeting nanomedicines are under preclinical investigation in fields such as tumor immunology.

At the interface of microbiota and immunity, nanomaterials can remodel microbial metabolism in an AHR-dependent manner to modulate immune responses. For instance, in ulcerative colitis, Portulaca oleracea L.-derived natural exosome-like nanoparticle maintain gut microbial diversity, promote the growth of Lactobacillus ventriosus, and elevate indole derivative levels. These metabolites activate AHR in CD4⁺ T cells, reprogramming them into double-positive CD4⁺CD8⁺ T cells, reducing pro-inflammatory cytokine levels, and alleviating colitis 324. Similarly, in a sepsis model, oral administration of manganese-doped carbon dots restores gut microbiota homeostasis (enriching Clostridium and Bacteroides) and enhances indole-3-propionic acid (IPA) production. IPA activates AHR in macrophages, inducing anti-inflammatory polarization and attenuating gut-lung axis-mediated lung injury 318.

The development of smart nanomaterials has further advanced precision AHR-targeted strategies. Researchers have engineered multiple TME-responsive delivery systems. MMP-2-Responsive Polypeptide Micelles: Self-assembling polypeptide micelles co-loaded with the AHR inhibitor CH223191 and anti-CD28 antibody are engineered with an MMP-2-sensitive sequence. Following tumor accumulation via the EPR effect, TME-enriched MMP-2 triggers micelle dissociation and controlled CH223191 release. Dual-SHRP reverses immunosuppression by inhibiting AHR activation while enhancing T-cell co-stimulation, synergistically amplifying antitumor immunity and suppressing metastasis 325. Dual-Responsive Liposomal Vesicles: This system comprises an MMP-2-cleavable mPEG shell and a GSH-responsive core co-loaded with the IDO1 inhibitor NLG919 and the chemotherapeutic agent 5-FU. Tumor-enriched MMP-2 promotes specific accumulation and cellular uptake, while intracellular GSH triggers drug release. 5-FU induces immunogenic cell death (ICD), activating the immune system, whereas NLG919 blocks IFN-γ-driven Kyn-AHR signaling, reversing IDO1-mediated immune tolerance. This design achieves a chemo-immunotherapeutic cycle amplification 326. TME-Responsive Nanomedicine: IDO1 inhibitor 1-MT and nitric oxide donor GSNO are co-loaded onto PEGylated HMnO₂ nanoparticles. The system responds to the acidic TME, releasing its payload to enhance tumor immunogenicity while inhibiting IDO1 expression to counteract Trp metabolism-induced immunosuppression, thereby remodeling the metabolic microenvironment and synergistically boosting antitumor efficacy 327.

In summary, nanomaterials leverage their functional versatility and TME-responsive properties to achieve multidimensional precision regulation of the AHR pathway—either indirectly via microbiota-metabolic intervention or directly through smart delivery systems. These strategies overcome the targeting limitations of conventional therapies and provide novel tools for reversing immune tolerance and remodeling the TME, advancing the clinical translation of AHR-targeted therapeutics.

## Conclusions and prospects

In summary, AHRs, a class of ligand-activated transcription factors, play complex and multifaceted roles in tumorigenesis, immune regulation, and microenvironment remodeling. The activation status of AHR and its downstream signaling network can profoundly influence tumor immune responses, microenvironment characteristics, and therapeutic sensitivity by modulating immune cell functions, metabolic reprogramming, and epigenetic remodeling. Specifically, AHR is a notable "double-edged sword" in tumor immunity: on the one hand, it can foster an immunosuppressive microenvironment by suppressing CTL activity, promoting Treg differentiation, and expanding myeloid-derived suppressor cells, thereby facilitating tumor immune escape; on the other hand, in specific ligand or cellular contexts, AHR may also enhance DC antigen-presenting capacity, or remodel antitumor immune responses through immunogenic cell death. This functional diversity is likely attributed to the tissue-specific distribution of AHR ligands, the selective recruitment of receptor-interacting proteins, and the dynamic regulation of epigenetic modifications. AHR-targeted therapies face several challenges, including the potential for off-target effects due to the high tissue specificity of AHR signaling; complications in drug design arising from the dual functionality of ligand-bound AHR, which acts both as a transcriptional activator and an E3 ubiquitin ligase component that mediates protein degradation; and the risk of resistance driven by dynamic signaling fluctuations and compensatory pathway activation within the TME.

Future research should focus on the following directions. First, single-cell multiomics technologies and spatial transcriptomics should be leveraged to dissect the spatiotemporally specific functional landscape of AHR in different tumor types, developmental stages, and TME subcompartments, and the molecular switches governing its transition between "protumor" and "antitumor" roles should be clarified. Second, we explore the interaction network between AHR and other epigenetic regulators (e.g., DNA methyltransferases and histone deacetylases) in detail to reveal their epigenetic‒metabolic synergistic mechanisms in tumor immunoediting. Third, novel AHR allosteric modulators or PROTAC degraders should be developed to achieve precise intervention in the AHR signaling pathway, while organoid models and patient-derived xenograft (PDX) platforms should be utilized to optimize preclinical evaluation systems. Additionally, the regulatory effect of the gut microbiota‒host metabolic axis on AHR activity warrants attention. For example, specific microbiota-derived Trp metabolites may remotely regulate antitumor immunity via AHR signaling, which provides a theoretical basis for microbiome intervention-based combination therapies. As the understanding of the AHR signaling network has increased, its translational potential in tumor immunotherapy has gradually emerged. By integrating multidisciplinary approaches such as chemical biology, immunometabolism, and computational biology, it is anticipated that future efforts will establish personalized treatment strategies based on AHR targeting to overcome the resistance bottleneck of existing tumor immunotherapies and to provide novel opportunities for improving patient prognosis.

## Figures and Tables

**Table 1:** Ligands of AHR.

Classification	AHR Ligand
Chemicals	• 2,3,7,8-tetrachlorodibenzo-*p*-dioxin (TCDD) 54 • Polychlorinated dibenzofurans^55^ • Polychlorinated biphenyl**s** 56 • Benzanthracenes 57 • 3-methylcholanthrene(3MC) 58 • AGT-5 59 • VAF347 60
Dietary	Indoles: • Indolo[3,2-b]carbazole (ICZ) 61 • Indole-3-carbinol (I3C) 62 • 3,3′-diindolylmethane (DIM) 63 Others: • Quercetin 64 • Galangin^65^ • Astaxanthin 66 • Indirubin 67 • Ethyl caffeate 68 • Wogonin 69
Host metabolism	Ryptophan metabolites: • Kynurenic acid 70 • Kynurenine 71 Others: • Bilirubin 72 • Biliverdin 72 • Prostaglandin PGG2 73 • Hydroxyeicosatrienoic acid ([12(R)-HETE]) 74
Bacterial metabolism	• Tryptamine 75 • Indoxyl sulfate 76 • 6-formylindolo[3,2*b*] carbazole (FICZ) 77 • Malassezin 61 • Urolithin A 78 • Vitamin B12 79 • Folic acid 79 • Vitamin K2 80

**Table 2:** Clinical trials related to AHR modulators

Registration number	Clinical trials	Diseases	Intervention
NCT04069026	A First-in-Humans Dose Finding Study for an Aryl Hydrocarbon Receptor Inhibitor (AHRi) in Patients with Advanced Cancer	Solid tumors	BAY 2416964
NCT04200963	A Phase 1a/b Study of IK-175 as a Single Agent and in Combination with Nivolumab in Patients with Locally Advanced or Metastatic Solid Tumors and Urothelial Carcinoma	Solid tumors	IK-175, nivolumab
NCT05472506	Oral AHR Antagonist in Combination with Nivolumab in Patients With PD-1 Resistant Metastatic or Recurrent Head and Neck Cancer	Head and neck squamous cell carcinoma	IK-175, nivolumab
NCT04999202	A Study to Learn How Safe the Study Drug BAY 2416964 (AHR Inhibitor) in Combination with the Treatment Pembrolizumab is, How This Combination Affects the Body, the Maximum Amount That Can be Given, how it Moves Into, Through and Out of the Body and Its Action Against Advanced Solid Cancers in Adults	Solid tumors	BAY 2416964, pembrolizumab
NCT04053387	Long Term Extension Study of Tapinarof for Plaque Psoriasis in Adults	Plaque psoriasis	Tapinarof cream
NCT06161415	Safety, Tolerability, and Distribution of Laquinimod Eye Drop: The LION Study (LION)	Uveitis	Laquinimod eye drops

## Abbreviations

3MC: 3-methylcholanthrene
AHR: Aryl hydrocarbon receptor
AD: Atopic dermatitis
AHRE: AHR response elements
AHRR: Aryl hydrocarbon receptor repressor
APC: Antigen-presenting cells
ARNT: Aryl hydrocarbon receptor nuclear translocator
BaP: Benzopyrene
BHLH-PAS: Basic helix-loop-helix/Per-Arnt-Sim
BMAL1: Basic helix-loop-helix ARNT like 1
Brg1: Brahma/SWI2-related gene 1
CA: Cinnamic acid
CRC: Colorectal cancer
CSC: Cancer Stem Cell
CUL4B: Cullin 4B
CYP1A1: Cytochrome P450 1A
DC: Dendritic cells
DIM: 3,3'-diindolylmethane
DNMT: DNA methyltransferases
DRE: Dioxin response elements
EMT: Epithelial-mesenchymal transition
ER: Estrogen receptor
ETC: Electron transport chain
FICZ: 6-formylindolo[3,2*b*] carbazole
HCC: Hepatocellular carcinoma
HDAC: Histone deacetylase
HIF: Hypoxia-inducible factor
HK2: Hexokinase 2
HNSCC: Head and neck squamous cell carcinoma
HSP90: Heat-shock protein90
I3A: Indole-3-aldehyde
ICZ: Indole-3-carbinol (I3C)
IDA: 3-indoleacrylic acid
IDO: Indoleamine-2,3-dioxygenase
IEL: Intestinal intraepithelial lymphocytes
IPA: Indole-3-propionic acid
IR: Ionizing radiation
Kyn: Kynurenine
lncRNA: Long noncoding RNA
MMP: Mitochondrial membrane potential
MTS: Mitochondrial targeting sequences
NSCLC: Non-small cell lung cancer
OCT4: Octamer-binding transcription factor 4
OXPHOS: Oxidative phosphorylation
PDAC: Pancreatic ductal adenocarcinoma
PMN: Premetastatic niche
PDX: Patient-derived xenograft
ROS: Reactive oxygen species
SCLC: Small cell lung cancer
SLE: Systemic lupus erythematosus
SOX2: SRY-box transcription factor 2
SRC: Steroid receptor coactivator
STAT1: Signal transducer and activator of transcription 1
TAM: Tumor-associated macrophage
TCDD: 2,3,7,8-tetrachlorodibenzo-p-dioxin
TDO2: Tryptophan 2,3-dioxygenase
Th17: T helper 17
TiPARP: TCDD-inducible poly-ADP-ribose polymerase
TME: Tumor microenvironment
Treg: Regulatory T-cell
Trp: Tryptophan
XAP2: X-associated protein 2
XRE: Xenobiotic response elements
